# Activity Determinants of Helical Antimicrobial Peptides: A Large-Scale Computational Study

**DOI:** 10.1371/journal.pone.0066440

**Published:** 2013-06-12

**Authors:** Yi He, Themis Lazaridis

**Affiliations:** Department of Chemistry, City College of New York, New York, New York, United States of America; University of Melbourne, Australia

## Abstract

Antimicrobial peptides (AMPs), produced by a wide range of organisms, have attracted attention due to their potential use as novel antibiotics. The majority of these peptides are cationic and are thought to function by permeabilizing the bacterial membrane, either by making pores or by dissolving it (‘carpet’ model). A key hypothesis in the literature is that antimicrobial and hemolytic activity correlate with binding affinity to anionic and zwitterionic membranes, respectively. Here we test this hypothesis by using binding free energy data collected from the literature and theoretical binding energies calculated from implicit membrane models for 53 helical AMPs. We indeed find a correlation between binding energy and biological activity, depending on membrane anionic content: antibacterial activity correlates best with transfer energy to membranes with anionic lipid fraction higher than 30% and hemolytic activity correlates best with transfer energy to a 10% anionic membrane. However, the correlations are weak, with correlation coefficient up to 0.4. Weak correlations of the biological activities have also been found with other physical descriptors of the peptides, such as surface area occupation, which correlates significantly with antibacterial activity; insertion depth, which correlates significantly with hemolytic activity; and structural fluctuation, which correlates significantly with both activities. The membrane surface coverage by many peptides at the MIC is estimated to be much lower than would be required for the ‘carpet’ mechanism. Those peptides that are active at low surface coverage tend to be those identified in the literature as pore-forming. The transfer energy from planar membrane to cylindrical and toroidal pores was also calculated for these peptides. The transfer energy to toroidal pores is negative in almost all cases while that to cylindrical pores is more favorable in neutral than in anionic membranes. The transfer energy to pores correlates with the deviation from predictions of the ‘carpet’ model.

## Introduction

Antimicrobial peptides (AMPs) are found in a wide variety of organisms such as plants, insects, and vertebrates, providing a host-defense mechanism against invading microbial species [1]–[4]. These peptides usually exhibit selectivity against prokaryotic pathogen cells over the host cells [5]–[7]. Some show selectivity for fungi, cancer cells and parasites [8], [9]. AMPs are thought to be less likely to elicit resistance than traditional antibiotics, and this gives them potential for clinical applications [10]. Some AMPs have been found to have intracellular targets, but the majority are thought to kill bacteria by disrupting the cell membrane [11]–[14] either by pore formation [15] or by detergent-like disintegration (the ‘carpet’ model)[16]. Even when AMPs target intracellular sites, they still have to cross the cell membrane. Experiments with several AMPs have shown their ability to translocate across cell and liposome membranes, a property they share with cell-penetrating peptides[17]. Thus, understanding the process of translocation and pore formation could have wide-ranging implications.

Despite the vast amount of biological and biophysical data collected on AMPs, unifying concepts are still lacking. It is not yet possible to look at the sequence and even the structure and thermodynamic properties of a peptide and predict whether it is antimicrobial or cell-penetrating. Some peptides are helical, others contain beta structures, and still others are unstructured. Even random copolymers that lack a regular folding pattern have been shown to possess antimicrobial activity[18]. Thus, secondary structure does not provide any guidance. Several bioinformatics methods have been proposed to identify and predict the activity of AMPs based on simple descriptors like hydrophobicity, amphipathicity, charge, or helicity [19]–[21]. They are partially successful [7], [22]–[27] but the lack of connection to a physical mechanism places limits in their applicability and ultimate utility.

An intuitive idea is that the biological activity of a peptide should be related to its affinity for the target membrane. Most, but not all, AMPs are cationic, and this provides an intuitive rationalization for their targeting bacteria: bacterial membranes are negatively charged, whereas the outer leaflet of eukaryotic membrane is neutral [7], [28], [29]. This hypothesis is supported by a lot of anecdotal evidence and has been stated most explicitly in recent work [30], [31]. If the ‘carpet’ model were universally valid, one would expect a perfect correlation of biological activity with membrane binding affinity. However, this correlation might break down in practice due to presence of other steps, such as membrane insertion and pore formation. For instance, the cationic melittin exhibits higher affinity for negatively charged membranes but its permeabilizing activity is higher for zwitterionic vesicles [32], [33]. A peptide could, in principle, bind very strongly (especially if inserted perpendicularly to the membrane surface) without causing any damage to the membrane or compromising its barrier properties[34]. Another reason for lack of correlation with membrane binding affinity could be the complexity of the biological process: for example, to get to the plasma membrane AMPs need to traverse either the outer membrane of gram negative bacteria or the cell wall of gram positive bacteria [35]. The activity of a peptide could be affected by these steps (if they are rate-limiting) rather than the interaction with the plasma membrane.

Recent work in our group showed that the hemolytic activity of a series of four peptides correlated with a theoretical estimate of the binding affinity to a zwitterionic bilayer [36]. The correlation was observed whether one used the binding energy to the planar bilayer, the binding energy to the pore, or the difference between the two. The goal of the present work is to perform a systematic, larger-scale test of this hypothesis. First we examine the correlation between experimental affinity and biological activity data across a wide variety of helical AMPs. Then we complement these data with computational studies using an implicit membrane model which can account for the effect of membrane surface charge[37]. Computational studies have the advantage that they are performed under the same conditions and provide structural information, within the uncertainties, of course, of the theoretical treatment. A set of 53 helical peptides that have known biological activities and 3D structures were considered and their interfacial binding energy to zwitterionic and anionic membranes was calculated by molecular dynamics (MD) simulations.

There are several challenges in the compilation of experimental data. One difficulty is that the reported binding free energies are not always comparable. Most studies analyze the data using the “single set of sites” model, i.e. treating the membrane as a receptor with many, equivalent, and independent binding sites. Others use a simple mass action law[30] while others use a partitioning formalism[38], [39]. The Seelig group reports free energies that have been corrected for generic electrostatic effects using the Gouy-Chapman theory[40]. To be comparable, data have to be converted to the same standard state (see Section S1). Biological activity data present even more severe challenges. The calculated MIC values are sensitive to a variety of conditions: the bacterial strain, the culture medium[41], the salt concentration[42], the bacterial concentration[43], etc. As a result, MIC values for the same peptide and for the same bacterium reported by different laboratories are often very different. For example, the MIC of magainin against E. Coli has been reported as 0.4∼2.8 µM[42] or 38 µM[44]. These uncertainties will introduce significant scatter in any attempt to correlate data on a large scale. However, some signal should still be visible.

We found that the surface charge difference between bacterial and eukaryotic membranes is indeed an important determinant of peptide selectivity: antibacterial activity correlates best with transfer energy to a membrane containing over 30% anionic lipid; hemolytic activity correlates best with transfer energy to a membrane containing 10% anionic lipid. Other physical descriptors, in addition to membrane binding energy, were also found to correlate with biological activity: surface area occupation of the peptide is an important descriptor for antibacterial activity; insertion depth is an important factor in hemolytic activity, while structural flexibility affects both antibacterial activity and hemolytic activity. The membrane surface coverage by many peptides at the MIC is estimated to be much lower than would be required by a ‘carpet’ mechanism. The peptides suggested in the literature to form pores are highly likely to have lower surface coverage than those suggested to act by the ‘carpet’ model. Using implicit membrane modeling, we were also able to calculate the transfer energy to pores. Formation of a toroidal pore is more likely than barrel-stave pore for most peptides. The deviation from predictions of the ‘carpet’ model was found to correlate with transfer energy to pores.

## Results and Discussion

### a) Configurations of antimicrobial peptides at the membrane interface

Antimicrobial peptides exhibit wide structural diversity. Even after limiting the range to helical peptides, the peptide can be as short as 13 residues (IsCT) or as long as 42 residues (moricin); it can be highly helical (δ-hemolysin) or mainly unfolded (SMAP29); it can also be highly linear (mastoparan M) or have multiple flexible hinges (moricin); the charge can range from -1 (alamethicin) to +12 (CAP18).

**Figure 1:**
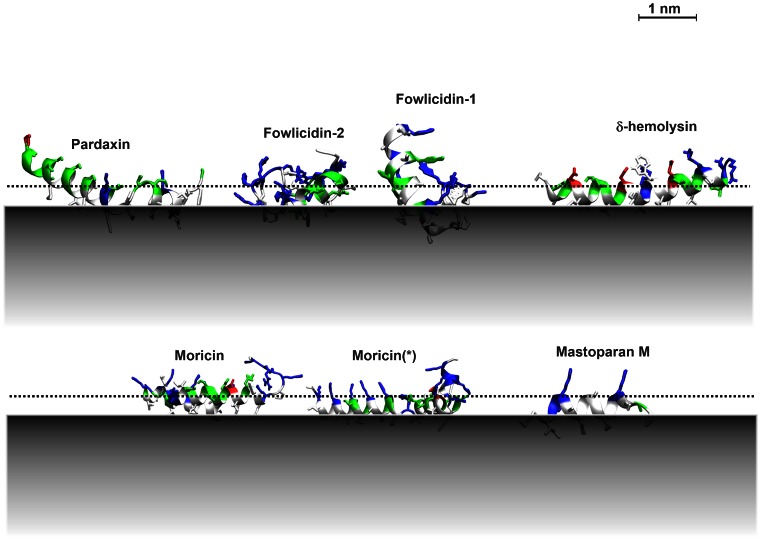
Typical conformations of antimicrobial peptides on anionic membranes. The lowest energy conformations in the last 2ns of 4 ns implicit simulations on 30% anionic membranes are shown. The grey areas indicate the hydrophobic core of the membrane. The dotted lines indicate the location of the phosphate groups. The colors on the peptides indicate the residue type: Red: acidic; Blue: basic; white: polar; green: uncharged polar. Moricin (*): an alternative conformation of moricin (1kv4) with the same total energy.

As a result, the behavior of the peptides at the membrane interface is quite diverse. Figure 1 shows several lowest energy conformations from the last 2ns of 4 ns of implicit membrane simulations on 30% anionic membranes, using the protocol described in the Materials and Methods section. The peptide can insert deep into the hydrophobic core (8 Å, alamethicin) or only penetrate shallowly (1 Å, such as fowlicidin-2); the tilt angle of the peptide can range from 80^°^ (pardaxin) to 120^°^ (such as fowlicidin-1). The structure can be quite rigid (rmsf < 0.9 Å, such as mastoparan M) or can exhibit large fluctuations (rmsf > 5 Å, such as moricin). The conformations on neutral membranes are similar, except the binding energy is less negative and the peptide inserts less deeply into the membrane.

### b) Correlation between theoretical transfer energy and experimental binding free energy

Thermodynamic data for membrane binding in the literature are reported in a variety of standard states. For comparison between them they need to be converted to the same standard state. Section S1 in the supplementary material describes how this is done and presents a compilation of data on AMPs.

The membrane binding free energy can be broken up into the following contributions (see Section S1): a) the change in average effective energy, Δ<W>, which includes intramolecular and solvation free energy changes, b) translational and rotational entropy changes, c) the free energy of folding, if the soluble form is disordered and the membrane-bound form has secondary structure, and d) the disaggregation free energy, if the peptide is aggregated in solution [45]. Using an implicit model it is relatively straightforward to obtain Δ<W>: for each peptide, we place it on the membrane or in water and calculate the difference in average effective energy in MD simulations. However, for peptides that are unfolded or aggregated in solution this simple approach is not sufficient.

For peptides that are disordered in solution and fold into a helix upon membrane binding, a useful approach might be to calculate the free energy of the transition from membrane-bound helix to a helix in solution. This value should be added to the free energy of helix unfolding in solution, which for these peptides should be a small number, not more than 2–3 kcal/mol [37]. The translational-rotational entropy of peptide adsorption to a membrane has been estimated as 1.3 kcal/mol [46] and is likely to be similar for different peptides. The effective energy of membrane binding of the helix is estimated from the average transfer energy, <ΔW>, obtained by averaging the difference in effective energy between membrane and aqueous phase for every conformation generated during the membrane simulation. <ΔW> has the added advantage of a lower statistical uncertainty than Δ<W>. According to this rationale, <ΔW> should be systematically more negative than the experimental binding free energy by a few kcal/mol.

We pursued both approaches and computed <ΔW> from 4-ns simulations on the membrane and Δ<W> from separate 100-ns simulations both on the membrane and in solution. The results are listed and compared with the experimental binding free energies in Table 1. <ΔW> is more negative than the ΔG in all cases except for Dermadistinctin K and mastoparan X. The values of Δ<W> are almost always smaller in magnitude than <ΔW> and in most cases closer to the values of ΔG. In the long simulations used to compute Δ<W>, we observed that most peptides maintained their initial structure on the membrane but became partially or completely unfolded in water. Only Dermadistinctin K and mastoparan X stayed mainly helical in water. This may indicate that, although these peptides are unstructured in solution [47], [48], the free energy of helix unfolding we neglected in <ΔW> may be smaller in these two cases than for the other peptides.

**Table 1:** Comparison of the experimental binding free energy with Δ<W> and <ΔW>.

Peptide	PDBid	Lipids	ΔG_c_^0^	<ΔW>	Δ<W>	Ref.
Alamethicin	1amt	DOPC	–5.77	–8.6±0.2	–8.2±0.4	[89]
Mastoparan X	2czp	POPC	–4.7	–4.2±0.8	–4.0±0.6	[17]
δ-hemolysin	2kam	POPC	–6.0	–12.6±0.5	–17.6±7.7	[30]
CM-15	2jmy	DMPC	–4.72	–8.3±0.9	–5.1±1.8	[90]
		DMPG	–5.49	–15.5±0.3	–14.1±3.2	
Dermadistinctin K	2k9b	egg PC	–3.97	–3.1±1.7	–2.5±3.1	[91]
LL-37	2k6o	SOPC	–6.16	–12.7±0.4	–1.8±3.8	[92]
		SOPC/POPG(7:3)	–8.79*	–19.1±1.9	–6.9±3.6	
Magainin	2mag	POPC	–3.7	–5.4±0.6	–1.8±3.1	[93]
		POPC:POPG(3:1)	–5.98	–10.7±1.0	–7.6±2.8	
Melittin	2mlt	DOPC	–5.1	–13.1±0.7	–5.4±1.2	[94]
		POPC/POPG(8:2)	–8.2	–18.6±2.5	–17.0±4.1	[95]
Pardaxin	1xc0	POPC	–6.21	–22.1±0.8	–7.0±2.3	[96]

* Interpolated from other anionic fractions.

**Figure 2:**
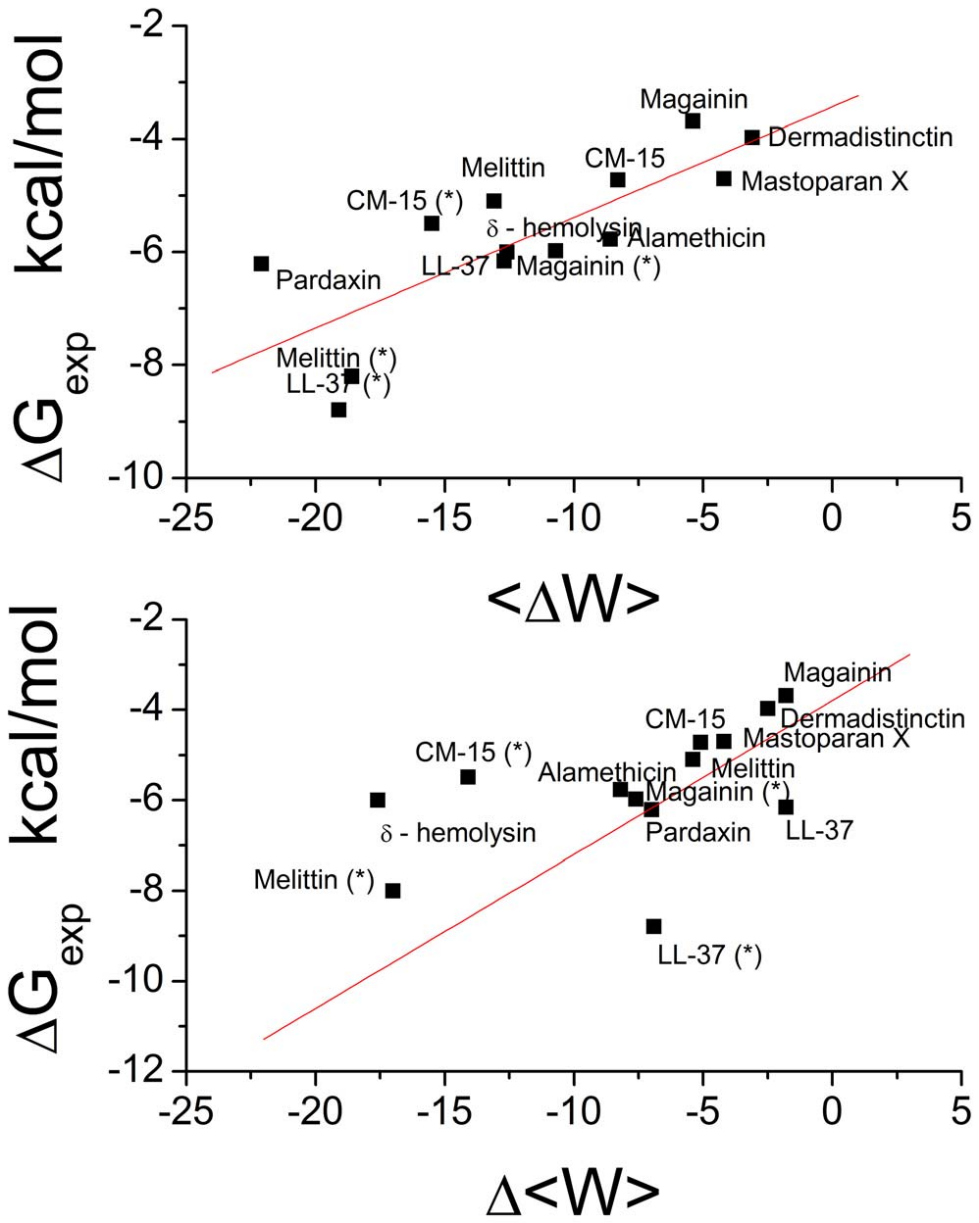
Correlation between theoretical binding energy and experimental binding energy ΔG_exp_. Binding energy values that are measured in anionic membranes are marked with (*). Upper panel, < Δ W>. Lower panel, Δ < W>.



Scatter plots that compare ΔG with < ΔW> and Δ<W> are shown in figure 2. A strong linear relationship can be found between <ΔW> and ΔG_exp_:

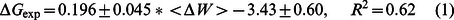



The correlation between ΔG and Δ<W> is much weaker (Fig. 2b). Extending some of the simulations up to 1000 ns did not improve the results significantly. Excluding the points that clearly do not fit (CM-15(*), Melittin(*), pardaxin), a linear relationship between Δ<W> and ΔG_exp_ can also be established but with higher uncertainty:
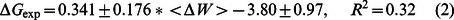



All-atom explicit simulations also exhibit significant deviations from experimental binding free energies. Several cases are listed in Table S4 and compared to transfer energies using the present implicit model. In some cases, the binding free energies calculated from explicit simulations are comparable to <ΔW>. In other cases, the <ΔW> from implicit simulations is closer to the experimental value than the explicit simulation result. As explained above, an important missing factor in these calculations is conformational entropy. For β antimicrobial peptides which have relative rigid structure, the transfer energy <ΔW> is closer to the binding free energy.

Even though not directly comparable with experimental binding free energy, <ΔW> can still be quite useful in practice thanks to its linear relationship with ΔG_exp_. In addition, calculating <ΔW> requires much less computing effort.

### c) Correlation between experimental membrane binding free energy and biological activity

**Figure 3:**
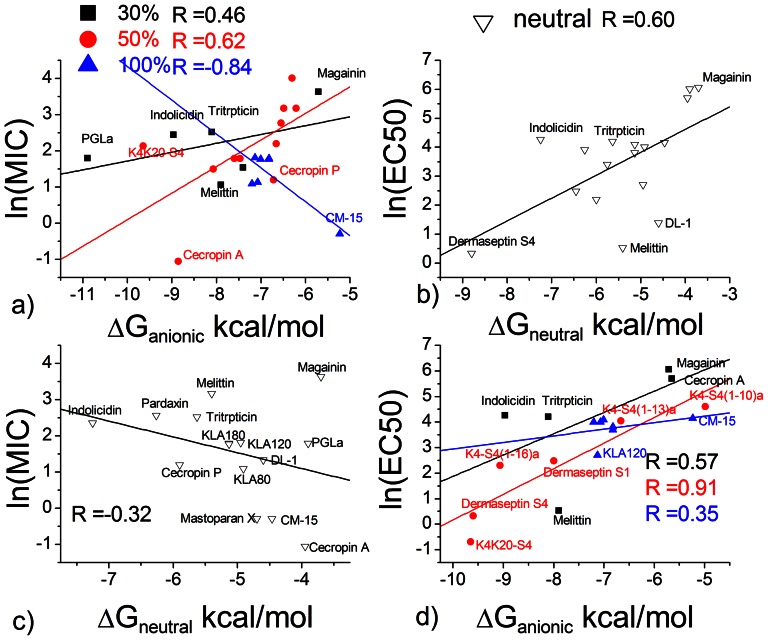
Correlation between experimental binding energy and biological activity. The lines are the best fit lines. a) ΔG_anionic_ in 25–100% anionic lipid and antibacterial activity against E. Coli. b) ΔG_neutral_ and hemolytic activity. c) ΔG_neutral_ and antibacterial activity. d) ΔG_anionic_ and hemolytic activity. The data points are chosen from Table S1. The unit of MIC and EC50 is µM.

To examine the relationship between membrane partition and biological activity, we collected from the literature minimal inhibitory concentrations (MIC) for antibacterial activity and 50% hemolysis concentrations (EC50) for hemolytic activity. Tables S2 and S3 list the collected data. Table S1 lists experimental binding free energies from the literature. Because concentrations are exponentially related to energies (see Eq. 14), we plot the natural log of MIC and EC50 against the binding energy to neutral or anionic membranes in Figure 3. There is considerable scatter in the data, but some correlations are visible. The linear correlation coefficient (R) between the ln(MIC) and binding energy to anionic membrane is 0.46 and 0.62 for anionic lipid fraction 25–30% and 50% respectively. The correlation for 100% anionic membrane, however, is negative largely due to a single data point: CM-15 is quite effective even though its membrane binding was measured to be rather weak. The binding energy to neutral membrane has a weak negative correlation (R = –0.32) with the ln(MIC).

The linear correlation coefficient between the ln(EC50) and neutral membrane affinity is 0.60. Similar correlation is observed between ln(EC50) and anionic membrane affinity (R =  0.57 and 0.91 for anionic lipid fraction 25–30% and 50% respectively).

### d) Correlation between theoretical transfer energy and biological activity

**Figure 4:**
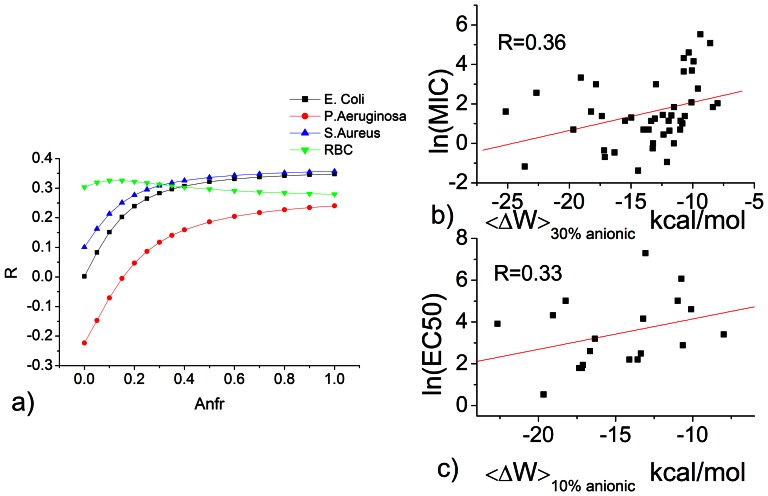
Correlation between transfer energy and biological effective concentrations. a) The effect of anionic lipid fraction (Anfr) on the correlation coefficient(S) between transfer energy <ΔW> and biological effective concentrations. b) ln(MIC) against E. Coli c). ln(EC50) against red blood cells. The <ΔW> and effective concentration values shown here are listed in Table S2.The unit of MIC and EC50 is µM. Red lines in b) and c) are the best fit lines.

For many peptides, the experimental binding free energy is not available, especially to anionic lipids. These are obtained here theoretically using an implicit membrane model. We ran MD simulations at different anionic fractions (anfr) and calculated the correlation coefficient (R) between <ΔW> and natural log of biological effective concentrations (figure 4a). Antibacterial activity against all three organisms correlates best with transfer energy to ≥ 30% anionic lipid membrane and quickly diminishes as the anionic fraction approaches 0%. The correlation to hemolytic activity is largely independent of anionic fraction; it is actually somewhat larger for 10% anionic fraction. It is difficult to say whether this slight increase in correlation is significant. It is worth noting, however, that the erythrocyte membrane contains around 30% phosphatidylserine (PS) in the inner leaflet[49]. Considering that the antimicrobial peptides can form pores, translocate through the membrane or induce lipid flip-flop in the membrane, it is conceivable that they could interact with the anionic lipids in the inner leaflet. In addition, the outer leaflet of erythrocyte membrane contains glycoproteins and glycolipids possessing anionic sialic acid groups [50], [51]. Specific scatter plots of the data are shown in figure 4 b&c. The linear correlation is slightly lower, due to the increased sample size and the difference between <ΔW> and ΔG, but still is statistically significant.

These data, together with those of the previous section lead to similar conclusions: a) there is a statistically significant correlation between binding energies and biological activities, b) electrostatic interactions are important for antimicrobial activity but much less so for hemolytic activity, and c) the scatter in the plots shows that membrane affinity is likely not the sole determinant of activity.

### e) Test of the ‘carpet’ model

**Figure 5:**
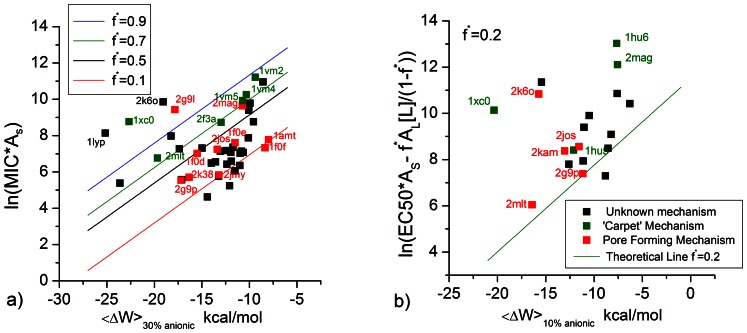
Biological activity of peptides plotted against theoretical transfer energy. a) activity against E. Coli. b) activity against red blood cells. The lines correspond to the theoretical expectation from the ‘carpet’ model for different value of the critical surface fraction covered. The red points are the peptides proposed to adopt the ‘pore forming’ mechanism. The green points are the peptides proposed to adopt the ‘carpet’ mechanism. The black points are the peptides that have unknown mechanism.

The ‘carpet’ model of AMP action proposes that peptides accumulate on the membrane surface until a critical point is reached at which the membrane disintegrates[16]. It has received considerable support from solid state NMR experiments [52]. It is possible, however, that these experiments detect a major, surface-bound fraction rather than a minor fraction that forms pores [53]. In this simple model, AMP activity depends on only two parameters: the membrane affinity of the peptide and the critical surface coverage that a certain type of membrane can withstand. The latter is not known, but it is reasonable to assume that it will fall in the range 50–90%. In Methods we derive a simple equation relating the MIC or the EC50 to these two parameters. If we insert Eq. 1 into Eq. 15 and Eq. 16, we obtain:
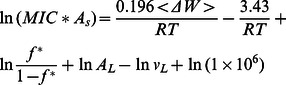
(3)

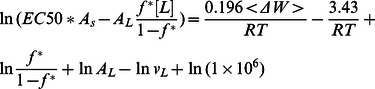
(4)


where the last term is a conversion factor fromµΜ to M. Figure 5a plots the calculated <ΔW> against ln(MIC*A_s_) and eq.3 with A_L_ = 65 Å and *v*_L_ = 0.76 M^−1^ [31] and different values of f^*^. The f^*^ = 0.9 line is close to the upper points in the plot, corresponding to peptides that are rather inactive compared to their computed binding affinity. A large number of points fall below the f^*^ = 0.5 line, i.e. they are more active than the ‘carpet’ model predicts. This can be an indication that these peptides act by a more efficient mechanism, for example pore formation. Figure 5b plots the <ΔW> against 
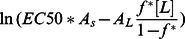
 assuming [L] = 20 µM [54]. Because when f* = 0.5–0.9, some peptides have
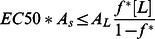
, we only plot the case of *f* ^*^ = 0.2. For many peptides large values of *f* ^*^ are simply not feasible under the conditions of the hemolysis experiments.

It is interesting to compare the position of each peptide in these graphs to experimental data suggesting their mechanism of action. In table S5, we collected information on the mechanism of various peptides suggested in the literature. It should be noted that most of these proclamations are based on circumstantial evidence that may be in conflict with another. For example, LL-37 was found to orient mainly parallel to membrane surface by Polarized ATR-FTIR spectroscopy[55] but partly perpendicular to the membrane surface by oriented CD spectroscopy[56]. Another example is pardaxin, which was originally referred to as a pore forming peptide[57] but more recent experiments suggested that it could adopt the ‘carpet’ mechanism in the presence of anionic lipids or cholesterol [58]–[60]. Sometimes, the conflict is reconcilable: magainin is suggested to act through the ‘carpet’ mechanism on eukaryotic cell membranes but not on bacterial membranes[61]. However, the stable pore formed in anionic membranes is preceded by formation of a large opening [62], similar to the ones thought to occur in the ‘carpet’ mechanism. Also, many of the suggestions are based on in vitro experiments that may not be entirely relevant to in vivo activities.

Keeping these caveats in mind, we marked the data points on figure 5 according to the proposed mechanism for each peptide. The peptides proposed to act by the ‘carpet’ mechanism are marked by green color and those proposed to be ‘pore forming’ are marked by red color. In general, the former peptides are less active and lie above the f^*^ = 0.5 line while most pore-forming peptides lie below this line. A few outliers can be observed, primarily gaegurin-4 (2g9l) and LL37 (2k6o). The mechanism of LL-37 (2k6o) is controversial: it has been suggested to form pores[56] but also to act as a ‘carpet’[55], [63]–[65].

### f) Correlation between transfer energy to pores and deviation from the ‘carpet’ model

Pore formation is a complex process with many contributions: peptide-lipid interaction, possibly peptide-peptide interaction, and the lipid deformation energy. But since forming a lipidic pore is energetically unfavorable[66], if a peptide tends to induce pore formation, the transfer energy of a single peptide to lipidic pores should be favorable. In previous work [36], four antimicrobial peptides were found to bind more strongly to toroidal pores than to the planar membrane. Here we extend this work to a larger number of peptides and to anionic pores [67]. We calculated the transfer energy of the peptides to pre-formed cylindrical and toroidal pores in neutral, 10% anionic and 30% anionic membrane of radius R_0_  = 13Å. The values are shown in Table S6. We observed that all peptides have favorable transfer energy to anionic toroidal pores. Transfer energy to cylindrical pores in anionic membranes is only favorable for a few peptides. In neutral membrane, the transfer energy to both pores is favorable for most peptides. Pardaxin (1xc0) has unfavorable transfer energy to any type of pore in neutral and 10% anionic membranes, even though a pore formation mechanism was proposed for it[57]. The peptide changed into a hairpin like structure in toroidal pores. It may be that the peptide adopts a specific structure that we have not been able to sample and that this is a metastable structure. Peptide-peptide interactions, which we haven’t taken into account in this study, may also contribute to stabilizing the pore [68].

**Figure 6:**
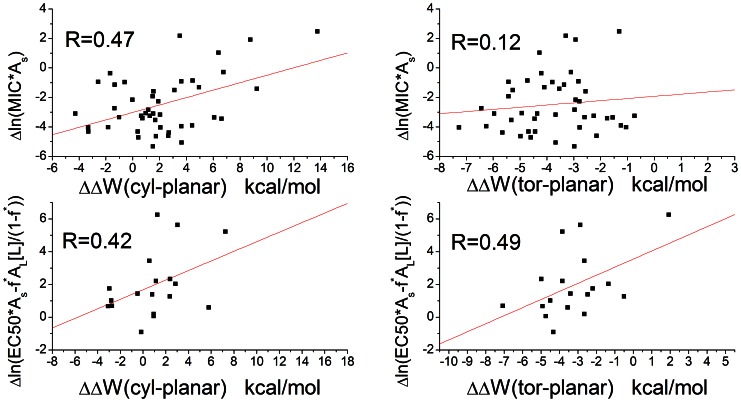
The correlation between transfer energy to pores and deviation of ln(MIC*A_S_) and
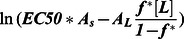
 from predictions of the ‘carpet’ model. cyl: the pore is a cylindrical pore with R = 13Å. tor: the pore is a toroidal pore with R = 13Å, K = 15Å, *h* = 0.6. The anionic fractions of the lipids are 10% and 30% respectively for ln(MIC*As) and 
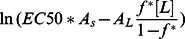
. The red lines are the best fitted lines. For 
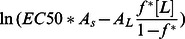
, [L] is assumed to be 20 µM and *f* ^*^ is assumed to be 0.2.

If pore formation makes the peptide more effective than the ‘carpet’ model would predict, we should observe that the reduction in ln(MIC*A_S_) and 
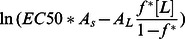
from the value expected for the ‘carpet’ model is positively correlated with the transfer energy to pores. Figure 6 shows that this indeed is the case: the more negative the ΔΔW, the more likely the ln(MIC*A_s_) or 
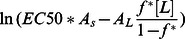
 to be lower than the values predicted by the ‘carpet’ model using *f* ^*^ = 0.9 or *f* ^*^ = 0.2 (to make sure all peptides have 
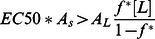
; similar correlation can be observed if *f* ^*^ = 0.9 was used for hemolytic activity but with much fewer data points). The correlation is significant (p-Value ≤ 0.1) except between Δln(MIC*A_S_) and ΔΔW_tor_. Interestingly, the transfer energy to cylindrical pores is more highly correlated with Δln(MIC*A_s_) than the transfer energy to toroidal pores, even though the latter is more favorable. This might be due to simplifications in the analysis, for example, toroidal pores induced by different peptides may have different curvature K_0_ and inhomogeneity factor *h*, but we are assuming they are constant in our simulations.

### g) Correlation between other physical descriptors and biological activity

Empirical correlations between peptide physicochemical properties and their antimicrobial activities have been investigated in the past [19], [21], [24], [69]–[71]. MD simulations offer additional descriptors that go beyond peptide sequence and secondary structure. The correlations between these descriptors and the biological effective concentrations are shown in Table 2. The Spearman correlation coefficient is used here because it is not affected by the actual mathematical relationship between effective concentration and descriptor. Because the MIC and EC50 increase when the activity of the peptide decreases, positive correlation in table 2 means unfavorable effect on the activity.

**Table 2:** Correlation between the biological effective concentrations and biophysical descriptors.

		MIC	EC50
		E.Coli.	Red Blood Cell
		N_pept_ = 44	N_pept_ = 19
n_charge_	The net charge of the peptides.	–0.46 (1.7E-03)	–0.13 (5.9E-01)
n_res_	Number of residues.	–0.36 (1.3E-02)	–0.23 (3.4E-01)
H	Hydrophobicity.	–0.09 (5.8E-01)	–0.08 (7.4E-01)
*µ_H_*	Hydrophobic moment.	–0.45 (2.4E-03)	–0.56 (1.3E-02)
Q_H_	Hydrophobic quadrupole moment.	–0.39 (8.2E-03)	–0.45 (5.1E-02)
ΔW	Transfer energy of peptide from water to membrane surface.	0.46 (1.7E-03)	0.30 (2.1E-01)
V	The immersed volume of peptide in the hydrophobic core of the membrane.	–0.15 (3.4E-01)	–0.22 (3.6E-01)
Depth	The penetration depth of peptide residue into the hydrophobic core. Calculated as the distance between the deepest residue and the membrane surface.	–0.14 (3.7E-01)	–0.48 (3.7E-02)
Tilt	The angle between peptide helix axis and membrane normal.	0.05 (7.6E-01)	0.29 (2.2E-01)
A_s_	Area of peptide occupied on the membrane surface.	–0.40 (7.6E-03)	0.12 (6.3E-01)
D_elec_	The electric dipole possessed by the peptide.	–0.25 (9.6E-02)	0.39 (0.9E-01)
Helix%	The percentage of helical structure.	0.25 (9.8E-02)	0.05 (8.3E-01)
n_helix_	The number of helical residues.	–0.13 (3.9E-01)	–0.25 (3.0E-01)
<n_hbond_>	The number of hydrogen bonds per residue that were formed inside the peptide.	–0.02 (8.8E-01)	–0.02 (9.2E-01)
n_hbond_	The number of hydrogen bonds.	–0.28 (6.5E-02)	–0.08 (7.4E-01)
rmsf	The maximum fluctuation of any residue in the simulation.*	–0.42 (4.3E-03)	–0.17 (4.9E-01)

The p values are given in the parenthesis (two tails T-test, probability that the actual correlation is below the given value). The descriptors were calculated from 30% anionic membrane simulations and 100% neutral membrane simulations respectively for correlating with MIC and EC50.

* If the average maximum fluctuation is used, the correlation is slightly lower.

Not surprisingly, the electrostatic interaction is crucial for selectivity: n_charge_ correlates significantly with antibacterial activity but not so well with hemolytic activity. Peptide size (n_res_) also correlates positively with the biological activity as previosly revealed[72]. As found by previous studies [24], [69], amphipathicity is an important factor determining the activity of the peptide, even surpassing in importance the overall hydrophobicity[73]. Another quantity can be determined is the hydrophobic quadrupole moment, which can serve as measure of imperfect amphipathicity [36]. We list the hydrophobic quadrupole moment in Table S7 and show several peptides with various hydrophobic quadrupole moments in Figure S1. The peptides that have high hydrophobic quadrupole moment have either a kinked structure or their nonpolar surface twined around the helix wheel. A peptide with such type of structure should have better contacts with curved surfaces such as toroidal pores. In contrast, peptides with low hydrophobic quadrupole moment have balanced polar and nonpolar residues. Indeed, our study indicates that the hydrophobic quadrupole is also an important determinant of biological activity.

MD simulation provides additional information that could help us identify important new activity determinants. As described in previous sections, the transfer energy ΔW correlates with both antibacterial and hemolytic activity. We observed that antibacterial activity and hemolytic activity are affected differently by membrane insertion, tilt angle and A_s_. The membrane insertion (both insertion volume V and insertion depth, the latter showing stronger correlation) positively correlates with the hemolytic activity but not antibacterial activity, consistent with the result for the tilt angle; the closer the tilt angle is to 90^o^, the less active the peptide is against erythrocytes. The surface area occupation A_s_ is positively correlated with antibacterial activity but not hemolytic activity. It has been found that the angle subtended by polar residues is important to both antimicrobial activity and hemolytic activity[74]. Decreasing the angle will increase both the insertion into the membrane and the membrane surface area occupation of the peptide. The fact that the electric dipole of the peptide is unfavorably correlated with hemolytic activity is also consistent with this trend: increasing the peptide dipole increases interactions with the head groups and enhances the binding to membrane interface while at the same time reducing the insertion into membrane hydrophobic core.

The effect of helicity and structural flexibility is sometimes ambiguous in experiments. Increasing the number of proline residues in a peptide significantly reduced its channel-forming activity and thus both antimicrobial and hemolytic activities [75]. The increased flexibility and reduced helicity of D-amino acid containing peptides normally abrogated their hemolytic activity but did not diminish their antibacterial activity [76]–[79]. Recent research however showed that proline-containing peptides have higher antibacterial activity and lower hemolytic activity [80]. We observed that the structural flexibility (reflected by the structural fluctuation in the MD simulations) is positively correlated with both antibacterial and hemolytic activities. This may be due to a lower entropy cost upon membrane binding, and thus a lower membrane binding free energy. Alternatively, flexibility might be required at subsequent steps, such as pore formation. The number of helical residues (n_helix_) and hydrogen bonds (n_hbond_) are positively correlated with the biological activities. However, increasing the helical percentage has a negative effect on the biological activities, possibly because structural flexibility is reduced. The average number of hydrogen bonds per residue (<n_hbond_>) also has no correlation with biological activity. Hemolytic activity generally correlates more with helix formation and antibacterial activity correlates more with structural flexibility.

Using the above data, we can also establish a relationship between biological activity and biophysical factors using multiple linear regression:
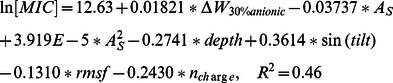
(5)

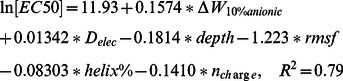
(6)


**Figure 7:**
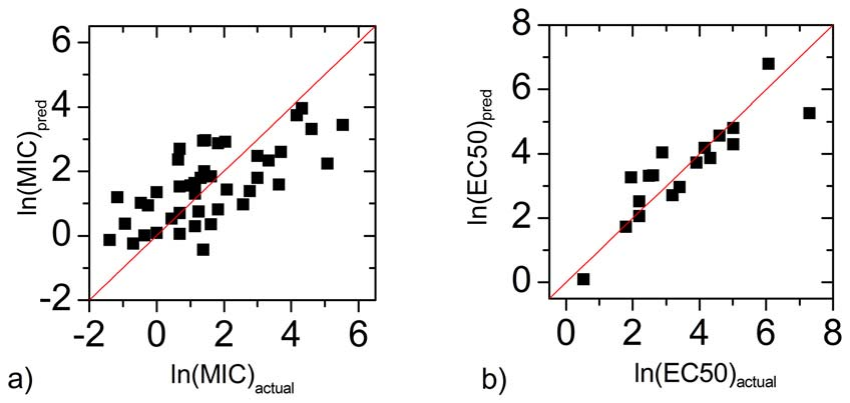
The predicted biological activity compared with the actual biological activity. a) the ln(MIC) calculated for E.Coli. membrane. b) the ln(EC50) calculated for Erythrocyte membrane.

In the above equation, adding or dropping a descriptor will reduce the correlation. The minimal set of parameters one needs to describe the biological activities is quite different for antibacterial activity and hemolytic activity. Predicted values using the above equations are plotted against the actual values in Figure 7. Compared to other QSAR studies, the correlation of predicted ln(MIC) with actual values is rather low. The mean square error is ± 1.5. Considering that the protocol of MIC/EC50 measurement allows a difference in concentration of 2 fold and the fact that our data points belong to different peptide families, the observed error is reasonable.

## Conclusions

Using experimental data and theoretical calculations we tested the hypothesis that antimicrobial and hemolytic activity correlate with the binding energy to the corresponding membranes. We found that such correlations do indeed exist, but they are weak, suggesting the involvement of factors other than membrane affinity. Electrostatic interactions of the peptides with the membrane do play an important role in the selectivity of the peptides. Interestingly, we found that the binding to a 10% anionic membrane correlates best with hemolytic activity, highlighting the importance of lipid translocation and small amounts of anionic lipids in the erythrocyte membrane.

Accurate calculation of membrane binding free energies is not possible at present, with either explicit or implicit solvation methods. However, the linear relationship between implicit membrane transfer energies and experimental binding free energy allowed us to estimate the membrane concentration at the MIC or EC50 for a large number of peptides. For many peptides this concentration was found to be much lower than one would expect for a ‘carpet’ mechanism. The previous finding that AMPs bind more strongly to toroidal pores than to the planar membrane is here confirmed for a larger number of peptides. Interestingly, the deviation of the MIC from the value expected for the ‘carpet’ model was found to correlate with the transfer energy from planar membrane to the pores.

The implicit membrane simulations can be used to obtain additional structural parameters and investigate their correlation with biological activity. Such parameters include the membrane surface coverage, the depth of insertion, the tilt angle and the flexibility. Deeper insertion into the membrane was found to correlate with hemolytic activity, while flexibility was found to correlate with antimicrobial activity. A new parameter that correlates with both activities is the hydrophobic quadrupole, a measure of imperfect amphipathicity.

## Materials and Methods

### a) Implicit membrane model

In the EEF1 [81] and IMM1[82] implicit solvation models, the effective energy (W_eff_) of a solute is the sum of the intramolecular energy of the solute (E) and its solvation free energy (ΔG^slv^). E is calculated from a modified version of the CHARMM19 force field, and ΔG^slv^ is obtained as the sum of atomic contributions, each calculated from a Gaussian solvent exclusion model.

The solvation parameters are expressed as a linear combination of the values for water and cyclohexane (mimicking hydrocarbon core):
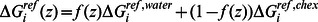
(7)


where *f*(z) is a switching function that depends on the position along the z axis (assumed to be the membrane normal):
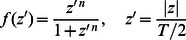
(8)


where T is the thickness of the membrane hydrophobic core. To describe the effect of anionic lipid on peptide binding, the electrostatic interaction between solute atom *i* and the membrane is calculated from:
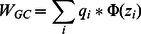
(9)


where φ(z) is obtained using the Gouy-Chapman theory[37].

The model was extended to pores in neutral [36], [83] and anionic membranes [67]. Two pore shapes are defined: cylindrical and toroidal. The first is a cylindrical hole inside the membrane filled with water. No head group is present at the pore wall so that this type of pore can mimic barrel-stave pores. In the later, the lipid head groups are bent to line the pore wall and its radius changes with the z coordinate:
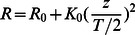
(10)


In anionic pores, the φ(z) was calculated by solving the Poisson-Boltzman equation. The head group dipoles were mimicked by a double layer of charges [67]. In this approach an additional parameter *h* is introduced as the homogeneity factor, which is the ratio of the head group density at the center of the pore to that on the planar membrane. All-atom simulations showed that this parameter is about 0.6 to 0.7 in pure toroidal pores.

### b) MD simulations

MD simulations of AMPs were conducted in planar membranes with anionic lipid fraction ranging from 0% to 100% in physiological salt solutions (0.1 M monovalent salt), using the membrane model presented in the previous section. The structures of the studied peptides were obtained from the PDB database. For NMR structures (most common) the lowest energy model for each peptide was selected and energy minimized using the steepest descent method for 300 steps. The peptides were placed on the membrane surface with their long axis parallel to the surface and rotated with the nonpolar residues facing the membrane. Four simulations with different random initial velocities were carried out. Each simulation consisted of 2 ns equilibration followed by 2 ns production. The latter was used for analysis. To calculate the Δ<W>, we simulated the peptide for 100 ns and used the last 50 ns for analysis. All simulations were carried out using the CHARMM program [84].

AMPs were also simulated inside cylindrical and toroidal pores using the standard IMM1 model for neutral pores [36] and the numerical Poisson-Boltzmann approach for anionic pores [67]. All pores have a radius of R_0_  =  13 Å. The toroidal pores have K_0_ =  15 Å and *h* = 0.6. Two anionic fractions were studied: 10% and 30%. The peptides were placed adjacent to the pore wall with their long axis parallel to the pore axis and their nonpolar residues facing the pore wall. Four simulations with different random initial velocities were carried out. Each simulation consisted of 3 ns equilibration followed by 3 ns production. The latter was used for analysis. The transfer energy to the pores, ΔΔW, was calculated as the difference between the <ΔW> to the pore and the <ΔW> to the planar membrane. To be consistent with the pore simulations, the <ΔW> to planar membrane was calculated using the Poisson-Boltzmann potential [67] instead of the Gouy-Chapmann theory.

### c) Calculation of physical descriptors from MD simulations

A list of the physical descriptors considered is shown in table 2. The first five are standard properties that can be obtained from the sequence.The overall hydrophobicity (H), hydrophobic dipole moment (µ_H_) and hydrophobic quadrupole moment (Q) of the peptides were calculated using the method and the consensus scale introduced by Eisenberg[85]. The original PDB structures were used to calculate these values. The net charge (n_charge_) was calculated as the sum of the charges of all ionizable groups at pH 7.

Additional descriptors were obtained from the MD simulations. The binding energy of the peptides to the membrane was estimated by the average effective energy change (ΔW_w->m_) upon transferring the peptide from the membrane surface (W_memb_) to water (W_water_), with conformation fixed. The immersed volume V was calculated by constructing a grid lattice around the peptide and counting the number of grids (0.1 Å) that are occupied by peptide atoms and at the same time are inside the membrane (z< T/2). The occupied area A_s_ was calculated as the volume of peptide inside a 1 Å slab centered at the membrane surface (z = T/2) divided by the slab thickness.

The tilt angle of the peptide was calculated as the angle between the principal axis of the peptide and the membrane normal (z axis). The insertion depth was calculated as the distance of the lowest residue of the peptide to the membrane surface. It is a negative value when the peptide has parts that are buried inside the membrane. The helix percentage was calculated using DSSP [86]. The electric dipole of the peptide can be calculated using the **coor dipole** command of CHARMM. Because the charged residues are neutralized in EEF1 and IMM1, we added back the charge to the charged residues before the calculation. The origin of the coordinate system was transferred to the peptide mass center when calculating the electric dipole. The number of hydrogen bonds n_hbond_ was calculated with cutoff distance 2.5 Å and cutoff angle 100^°^. The maximum fluctuation of the peptide (rmsf) is defined as the maximum value of the root mean square fluctuation of each residue.

### d) Data set

For comparison between experimental free energy and theoretical binding energy, 11 peptides were simulated and their ΔW was calculated. Experimental binding free energies (ΔG) were collected from the literature and summarized in Table S1. All binding free energy values were converted to the molarity standard state (ΔG_c_^0^, see Section S1).

The study was then extended to a total of 53 helical or partially helical peptides with known PDB structure selected from the APD database[87]. Large antimicrobial peptides (>50 residues) and beta structured peptides were not included in this study to avoid the complexity of additional structural variables. The activities of the peptides against *E. Coli*, *P. Aeruginosa* (typical Gram-negative bacteria), and *S. Aureus* (typical Gram-positive bacterium) were collected from literature data. These three organisms are most commonly used for measuring antimicrobial activity. A table listing the PDB id and activity of studied peptides can be found in the supplementary material (Table S2), where peptides are grouped by their origin and family. All the data collected come from experiments with standard or a variation of broth microdilution assay[88]. However, different colony formation units (CFU) are normally used in different studies so we listed the CFU conditions together with the MIC values in table S2. Data reported inµg/ml were converted to µM.

For comparing hemolytic activity, the peptide concentrations required to generate a certain extent of hemolysis are also listed in Table S2. Although the hemolytic assays were carried out in a standardized way, the incubation time and cell suspension concentration vary in different studies. These conditions are also listed in Table S2. Only the concentration to generate 50% hemolysis (EC50) was used to determine the relationship between hemolytic activity and peptide physical descriptors.

### e) Relationship between MIC and membrane binding affinity in the ‘carpet’ model

The MIC is the lowest overall peptide concentration at which bacterial growth ceases. If this is related to actions of the peptide on the membrane, the critical factor is the concentration of the peptide on the membrane. The higher the membrane binding affinity, the higher this concentration will be. The link between MIC and peptide concentration on the membrane has been worked out by Melo et al. [31]:
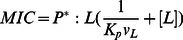
(11)


where P*:L is the critical molar ratio of membrane bound peptide to lipid, *K_p_* is the partition coefficient, and *v_L_* is the molar lipid volume. In a typical antibacterial assay, [L] is around 2 nM[54] to 58 nM[31] but the MIC is of the order ofµM and P^*^:L is in the range of 1∶10 to 1:100; [L] is thus negligible. Because ΔG_c_^0^ =  –RTln([P_L_] /[P_w_]), we can expand the previous equation into:
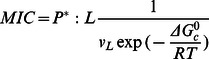
(12)


This is equivalent to assuming that the unbound peptide concentration is equal to the MIC. In the ‘carpet’ model, it is reasonable to assume that the membrane loses its integrity when the peptide covers a critical fraction (f^*^) of area of the lipids. *f* can be calculated as:
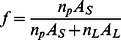
(13)


Thus the critical P^*^:L is:
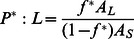
(14)


and 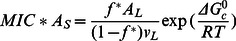
(15)


For the ‘carpet’ model, we expect f ^*^ to be a constant value. In implicit simulations, A_s_ can be easily obtained and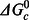 can be estimated from the transfer energy ΔW (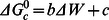, see Results).

Hemolysis assays are done at much higher lipid concentrations(20 µM [54] to 89 µM [31]) and [L] in Eq. 11 cannot be neglected. In this case we obtain:
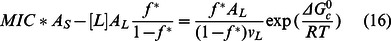



## Supporting Information

Figure S1 **Conformations of various antimicrobial peptides with their hydrophobic quadrupole moments.**
(PDF)

Table S1 **Compilation of experimental membrane binding free energies of AMPs, in the molarity standard state.**
(PDF)

Table S2 **Theoretical transfer energies and biological activities of the peptides.**
(PDF)

Table S3 **Biological activity of the peptides that do not have PDB structures.**
(PDF)

Table S4 **Comparison of the free energy from explicit simulations with experimental binding free energy and transfer energy <ΔW> from IMM1.**
(PDF)

Table S5 **List of peptide mechanism from literature.**
(PDF)

Table S6 **Transfer energy from planar membrane to pores.**
(PDF)

Table S7 **Hydrophobic quadrupole moment of studied peptides.**
(PDF)

Section S1 **Standardization of the membrane binding free energies.**
(PDF)
