# Supplementary material for: Activity Determinants of Helical Antimicrobial Peptides: A Large-Scale Computational Study
Source: PLoS One. 2013 Jun 12;8(6):e66440. doi: 10.1371/journal.pone.0066440 (PMC3680375; doi:10.1371/journal.pone.0066440)
Supplement: Figure S1 — Conformations of various antimicrobial peptides with their hydrophobic quadrupole moments. (PDF) [file pone.0066440.s001.pdf]

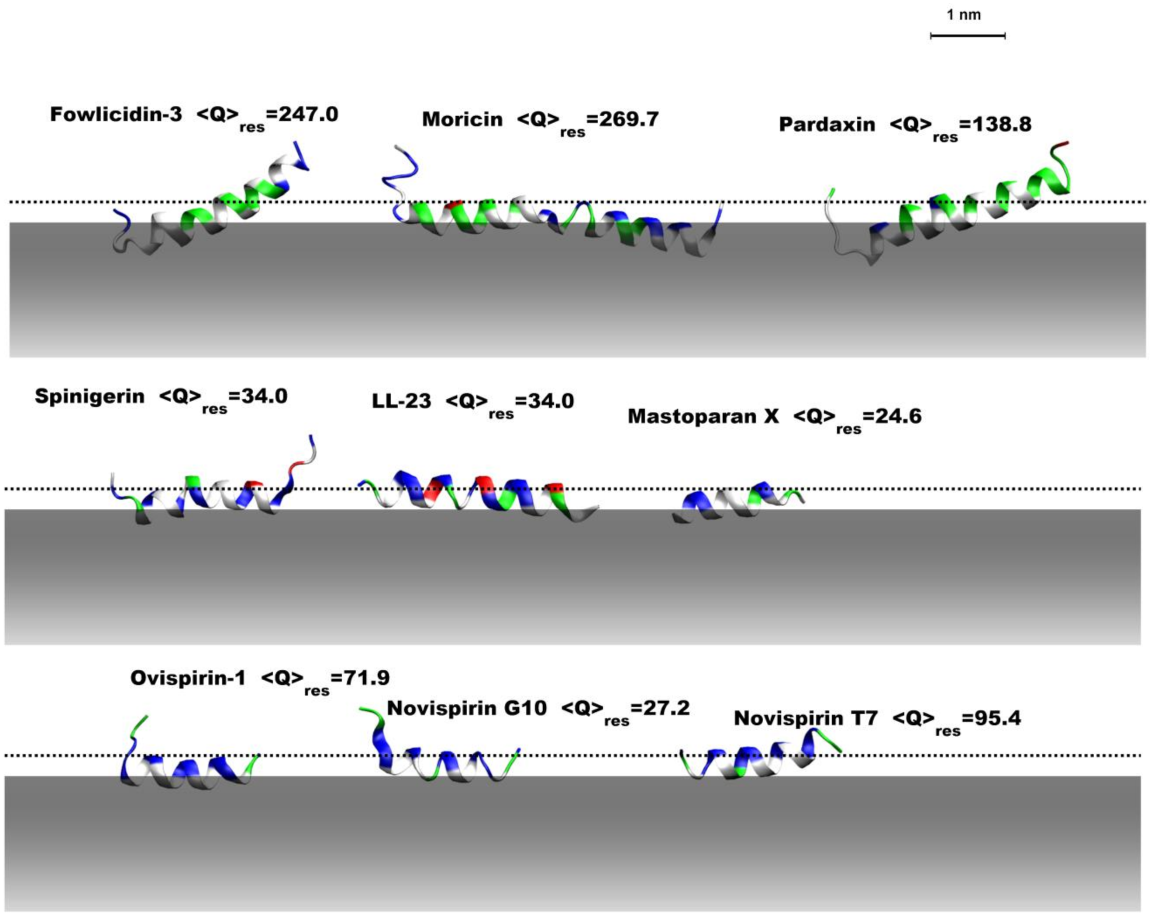

Figure S1. Conformations of various antimicrobial peptides with their hydrophobic quadrupole moments.

The lowest energy conformations in the last 2ns of 4 ns implicit simulations on 30% anionic membrane are shown with their corresponding $<Q>_{res}$. The grey areas indicate the hydrophobic core of the membrane. The dotted lines indicate the location of the phosphate groups. The colors on the peptides indicate the residue type: Red: acidic; Blue: basic; white: polar; green: uncharged polar.
