# Supplementary material for: Activity Determinants of Helical Antimicrobial Peptides: A Large-Scale Computational Study
Source: PLoS One. 2013 Jun 12;8(6):e66440. doi: 10.1371/journal.pone.0066440 (PMC3680375; doi:10.1371/journal.pone.0066440)
Supplement: Table S1 — Compilation of experimental membrane binding free energies of AMPs, in the molarity standard state. (PDF) [file pone.0066440.s002.pdf]

Table S1. Compilation of experimental membrane binding free energies of AMPs, in the molarity standard state.

The listed experiments are carried out in temperature close to room temperature. Unless specifically stated, the room temperature is assumed to be 25 ℃. a-f, the conversion and correction done to the peptides. No conversion is needed for those articles reporting ΔG_c (ref7, ref 22 and ref 24).

a. Converted from ΔG_{app}. With Gouy-Chapman correction, where in the parenthesis is the effective charge of the peptide and after the slash is the energy value with electrostatic interaction added back.
b. Converted from ΔG_{app}. The ΔG_{app} is measured at low peptide/lipid ratio. If present, the value inside the parenthesis is the peptide concentration at which the K_{app} was measured.
c. Converted from $\Delta G_{a,}$ which is independent of peptide concentration. ΔG_c= RTln($K_d v_L$).
d. Converted from ΔG_a but considering the binding site size. ΔG_c= RTln($nK_d v_L$). The binding site size n is in the parenthesis.
e. Converted from $\Delta G_{x.}$
f. Monolayer correction applied, where the peptide is considered to bind only to the outer leaflet of the membrane (the effective lipid volume $V'_L=0.6V_L$).
g. Interpolated.

In red font are the data used in Figure 3.

Peptide	Sequence	Method	Membrane	Binding Energy ΔG_c^0 (kcal/mol)	Original Data	Ref
Magainin	GIGKFLHSAKKFG KAFVGEIMNS-NH2	ITC	POPC/POPG(75:25)	-2.54 (3.7-3.8)/-7.50[a]<br>-5.72 (1μM)[b]	K_{app}=55.5 M^{-1}<br>K_{app}=1.2x10^{4} M^{-1}	[1]
		ITC	POPC SUV	-4.74[a]	K_{app}=2000 M^{-1}<br>under 30 ℃	[2]
		ITC	POPC/POPG 3:1<br>LUV<br>SUV	 -3.14/-8.16[a]<br>-2.64/-7.65[a]	under 45 ℃<br>K_{app}=110 M^{-1}<br>K_{app}=50 M^{-1}	[3]
		ITC	POPC/POPG (3:1)<br>SUV<br>POPC SUV	-6.0[b]<br><br>-3.7[b]	K_{app}=2x10^{4} M^{-1}<br>K_{app}=400 M^{-1}<br>under 23 ℃	[4]
		CD titration	POPC:POPG(3:1)	-5.98(0M)[b]	K_{app}=2x10^{4} M^{-1}	[5]
		Fluorescence Kinetics	POPC:POPG(1:1)<br>POPC:POPG(7:3)<br>POPC:POPG(8:2)<br>POPC:POPG(9:1)<br>POPC:POPG(10:0)	-7.99[c]<br>-6.23[c]<br>-5.56[c]<br>-4.84[c]<br>-3.30[c]	K_d=1.8 μM<br>K_d=35 μM<br>K_d=110 μM<br>K_d=370 μM<br>K_d=5000 μM	[6]
I6A8L15I17-M2a	GIGKFIHAAK KFGKLFIGEI MNS(NH2)	ITC	POPC SUV	-5.6[a]	K_{app}=7700 M^{-1}<br>under 30 ℃	[2]
I6V9W12T15I17-M2a	GIGKFIHSVKKWG KTFIGEI MNS(NH2)	ITC	POPC SUV	-6.1[a]	K_{app}=20000 M^{-1}<br>under 30 ℃	
I6L15-M2a	GIGKFIHSAKKFGK LFVGEIMNS-NH2	CD titration	POPC:POPG(3:1)	-6.11(0M)[b]	K_{app}=25000 M^{-1}	[5]
L2R11A20-M2a	GLGKFLHSAKRFG KAFVGEAMNS-NH2		POPC:POPG(3:1)	-5.40(0M)[b]	K_{app}=7400 M^{-1}	
I6A8L15I17-M2a	GIGKFIHAAKKFG KLFIGEIMNS-NH2		POPC:POPG(3:1)	-6.96(0M)[b]	K_{app}=105000 M^{-1}	
PGLa	GMASKAGAIAGKI AKVALKAL-NH2	ITC LUV	POPC/POPG(3:1)<br>POPC	-4.5(5)/-11.1[a]<br>-4.1[a]	K_{app}=1500 M^{-1}<br>K_{app}=800 M^{-1}<br>under 30 ℃	[2]

(continued in next page)

(Continued from previous page)

Peptide	Sequence	Method	Membrane	Binding Energy ΔG_c^0(kcal/mol)	Original Data	Ref
Melittin	GIGAVLKVLTTGL<br>PALISWIKRKRQQ-<br>NH_2	Surface potential measurement	Lethicin	-7.55(10mM Salt)[f]<br>-7.96(100mM Salt)[f]		[7]
		CD	POPC/POPG(9:1)<br><br>POPC/POPG(8:2)	-6.5(1.9)/-7.7 [a,f]<br>-6.3 (0.77μM)[b,f]<br>-6.5/-8.2[a,f]	K_{app}=4.5x10^4 M^{-1}<br>K_{app}=3.19x10^4 M^{-1}<br>K_{app}=4.5x10^4 M^{-1}	[8]
		Fluorescence NBD	POPC LUV_{200}<br>LUV_{100}<br>SUV<br>DOPC LUV_{200}<br>LUV_{100}	-4.92(2.2)[a,f]<br>-4.82(1.5) [a,f]<br>-5.16(1.2) [a,f]<br>-4.82(1.6) [a,f]<br>-5.01(1.3) [a,f]	K_{app}=6x10^3 M^{-1}<br>K_{app}=5x10^3 M^{-1}<br>K_{app}=9x10^3 M^{-1}<br>K_{app}=5x10^3 M^{-1}<br>K_{app}=7x10^3 M^{-1}<br>under 30 ℃	[9]
		Ultrafiltration	EPC<br>EPC:PS(85:15)	-5.1 [e,f]<br>-6.4 [e,f]	ΔG_x=-7.6 kcal/mol<br>ΔG_x=-8.9 kcal/mol	[10]
		Ultrafiltration	DOPC	-5.1 [e,f]	ΔG_x=-7.6 kcal/mol	[11]
		CD	 POPC/POPG(10:0)<br>POPC/POPG(9:1)<br>POPC/POPG(3:1)<br>POPC/POPG(1:1)	 -3.7[e,f]<br>-4.3(1.0)/-5.5 [e,f]<br>-4.62/-6.0 [e,f]<br>-5.0/-6.4 [e,f]	ΔG_x= kcal/mol<br>-6.2<br>-6.8/-8.0<br>-7.12/-8.5<br>-7.5/-8.9	[12]
Pardaxin	GFFALIPKIISSPLF<br>KTLLSAVGSALSSS<br>GGQE	Fluorescence NBD-label	POPC<br>LPS	-6.21 [b,f]<br>-7.1[c]	K_{app}=3.3x10^4 M^{-1}<br>K_d= 8.5 μM	[13,14]
Dermaseptin	GLWSKIKAAGKEA<br>AKAAAKAAGKAA<br>LNAVSEAV	Fluorescence NBD-label	PC<br>PC/PS(1:1)	-5.28 [b,f]<br>-6.12 [b,f]	K_{app}=6.6x10^3 M^{-1}<br>K_{app}=2.8x10^4 M^{-1}	[15]
Dermaseptin S1	ALWKTMLKKLGT<br>MALHAGKAALGA<br>AADTISQGTQ	Surface Plasmon Resonance	PC<br>PC/PA(1:1)	-6.47 [c,f]<br>-8.0 [c,f]	K_d=14.29 μM<br>K_d=1.01 μM	[16]
Dermaseptin S4	ALWMTLLKKVLK<br>AALNAVLGANA		PC<br>PC/PA(1:1)	-8.86 [c,f]<br>-9.64 [c,f]	K_d=0.25 μM<br>K_d=0.067 μM	
K4K20-S4	ALWKTLLKKVLK<br>AAAKAALKAVLV<br>GANA		PC/PA(1:1)	-9.68 [c,f]	K_d=0.0625 μM	
K4-S4(1-16)a	ALWKTLLKKVLK<br>AAAK-NH2		PC/PA(1:1)	-8.10 [c,f]	K_d=0.91 μM	
K4-S4(1-13)a	ALWKTLLKKVLK<br>A-NH2		PC/PA(1:1)	-6.68 [c,f]	K_d=10 μM	
K4-S4(1-10)a	ALWKTLLKKV-<br>NH2		PC/PA(1:1)	-5.01 [c,f]	K_d=167 μM	
DD K	GLWSKIKAAG-<br>KEAAKAAGKAAL<br>NAVSEAV-NH_2	ITC	PC-LUVs	-3.97 (9.7) [d,f]	K_d=100 μM	[17]

(continued in next page)

(Continued from previous page)

Peptide	Sequence	Method	Membrane	Binding Energy ΔG_c^0 (kcal/mol)	Original Data	Ref
CecropinP	SWLSKTAKKLENS AKKRISEGIAIAIQ GGPR	Fluorescence NBD	PC<br>PC/PS(1:1)	-5.98[b,f]<br>-6.78[b,f]	K_{app}=3.1x10^3 M^{-1}<br>K_{app}=1.2x10^5 M^{-1}	[18]
CecropinA	KWKLFKKIEKVGQ NIBDGIIKAGPAVA WGQATQIAK-NH2	Fluorescence kinetics	POPC:POPG(1:1)<br>POPC:POPG(7:3)<br>POPC:POPG(8:2)<br>POPC:POPG(10:0)	-8.88 [c,f]<br>-5.66 [c,f]<br>-5.03 [c,f]<br>-3.95 [c,f]	K_d= 0.24 μM<br>K_d= 56 μM<br>K_d= 270 μM<br>K_d=1000 μM	[19]
		Fluorescence Trp	POPC:POPA(8:2)	-5.92 (27.8) [d,f]	K_d=1.28 μM	[20]
δ -lysin	formyl- MAQDIISTIGDLVK WIIDTVNKFTKK	Fluorescence Kinetics	POPC	-6.0 [c,f]	K_d=30 μM	[21]
DL-1<br>δ –lysin D->K	formyl- MAQKIISTIGKLVK WIIKTVNKFTKK		POPC	-4.5 [c,f]	K_d=400 μM	
DL-2a	formyl- LAADLLAALGDLA KWLLDALAKAAK K		POPC	- 4.9 [c,f]	K_d=200 μM	
DL-2b	formyl- LAADLLAALGDLL KWLLDALAKLAK K		POPC	- 3.2 [c,f]	K_d=3400 μM	
CE-1	KWKLLKKLEKAG AALKEGLLKAGPA LALLGAAAALAK- NH2		POPC	- 4.5 [c,f]	K_d=400 μM	
CE-2	KWKLLKKLEKAG AALKEGLLKAGPA LALLGAAAALAK- NH2		POPC	- 3.0 [c,f]	K_d=4700 μM	
MG-1	GILKFLESAKKWL EAFLAEIMNS		POPC	-4.9 [c,f]	K_d=200 μM	
MG-2	GLGKLLHAAKKL GKAWLGELLAA		POPC	-3.9 [c,f]	K_d=1100 μM	
Tp10W	AGWLLGKINLKAL AALAKKIL-NH2	Fluorescence Kinetics	POPC	- 5.1 [c,f]	K_d=140 μM	[22]
Tp10W-COO	AGWLLGKINLKAL AALAKKIL		POPC	- 4.9 [c,f]	K_d=200 μM	
Tp10-7MC	AGYLLGK(- 7MC)INLKALAALA KKIL-amide		POPC	- 6.3 [c,f]	K_d=20 μM	
mastoparan X	INWKGIAAMAKKL L-NH2		POPC	-4.7 [c,f]	K_d=300 μM	

(continued in next page)

(Continued from previous page)

Peptide	Sequence	Method	Membrane	Binding Energy<br>ΔG_c^0 (kcal/mol)	Original<br>Data	Ref
Alamethicin	AibPAibAibAibAibQ<br>AibVAibGLAibPVAi<br>bAibEQPhl	EPR	DOPC	-5.77[f]	K_c=17100	[23]
Gramicidin S<br>14dK4	cyclo[VKLdKVdYPL<br>KVKLdYP]	ITC LUV	POPC/POPG(3:1)<br>POPC/POPS(3:1)<br>POPG<br>POPS<br>POPC	-6.2 (11.1) [d]<br>-6.5 (14.3)[d]<br>-8.3 (5) [d]<br>-7.6 (5.3)[d]<br>-2.0 (166.7)[d]	K_d=3.1 μM<br>K_d=1.7 μM<br>K_d=22 μM<br>K_d=62 μM<br>K_d=278 μM	[24]
CM15	KWKLFKKIGAVLK<br>VL	Tryptophan<br>time-resolved<br>fluorescence	DMPC<br>DMPG	-4.72<br>-5.49	$K_c=2.9\times10^3$<br>$K_c=1.06\times10^4$	[25]
		EPR	POPE/POPG(8:2)	-4.86[b,f]	$K_{app}=0.28\times10^4$ M^{-1}	[26]
LL37(F27W)	LLGDFFRKSKEKIG<br>KEFKRIVQRIKDFL<br>RNLVPRTES	Tryptophan<br>fluorescence	SOPC<br>SOPCPOPG(9:1)<br>SOPCPOPG(8:2)<br><br>SOPCPOPG(6:4)	-6.16[c,f]<br>-8.44[c,f]<br>-8.58[c,f]<br>-8.79[c,f,g]<br>-9.29[c,f]	K_d=23.8 μM<br>K_d=0.51 μM<br>K_d=0.40 μM<br><br>K_d=0.12 μM	[27]
Indolicidin	ILPWKWPWWPWR<br>R-NH 2	ITC<br>LUV	POPC<br>E Coli lipid	-7.26[c,f]<br>-8.98[c,f]	ΔG_a=-7.4 kcal/mol<br>ΔG_a=-9.12 kcal/mol	[28]
		Reverse HPLC<br>LUV	POPC<br>POPG	-6.3[e,f]<br>-9.0[e,f]	ΔG_x=-8.8 kcal/mol<br>ΔG_x=-11.5 kcal/mol	[29]
Tritrpticin	VRRFPWWWPFLR<br>R-COO^-	ITC<br>LUV	POPE/ POPG(7:3)<br>POPC<br>E Coli lipid	-8.59 [c,f]<br>-5.63 [c,f]<br>-8.14 [c,f]	ΔG_a=-8.73 kcal/mol<br>ΔG_a=-5.77 kcal/mol<br>ΔG_a=-8.28 kcal/mol	[28]
Tritrp1	VRRFPWWWPFLR<br>R-NH2		POPE/ POPG(7:3)<br>POPC	-9.86 [c,f]<br>-6.75 [c,f]	ΔG_a=-10.0 kcal/mol<br>ΔG_a=-6.89 kcal/mol	
Tritrp2	V**KK**FPWWWPFL**K**<br>**K**-NH2		POPC	-5.28 [c,f]	ΔG_a=-5.42 kcal/mol	
Tritrp3	VRRF**A**WWW**A**<br>FLRR-NH2		POPE/ POPG(7:3)<br>POPC	-9.09 [c,f]<br>-6.99 [c,f]	ΔG_a=-9.23 kcal/mol<br>ΔG_a=-7.13 kcal/mol	
LAP1	QPEWFKARRWQW<br>RMKKLGA	Fluorescence<br>Trp	DMPC:DMPG 1:1	-6.47 (19.5) [d,f]	K_d=0.728 μM	[30]
LAP2	TISQPEWFKARRW<br>QWRMKKLGA			-6.53 (22.0) [d,f]	K_d=0.581 μM	
LAP3	TISQAEWFKARRW<br>QWRMKKLGA			-6.19 (21.0) [d,f]	K_d=1.09 μM	
LAP4	TASQAEWFKARR<br>WQWRMKKLGA			-6.28 (21.8) [d,f]	K_d=0.898 μM	
LAP5	EWFKARRWQWR<br>MKKLGA			-7.63 (15.4) [d,f]	K_d=0.129 μM	
LAP6	EWFKARRWGWR<br>MKKLQA			-7.45 (15.4) [d,f]	K_d=0.175 μM	
KLA80	KLALKLALKWAK<br>LALKAA	CD titration	POPC<br>POPG	-4.91[b,f]<br>-7.20 [b,f]	K_{app}=5100 M^{-1}<br>$K_{app}=2.5\times10^5$ M^{-1}	[31]
KLA100	KLLAKAAKKWLL<br>LALKAA		POPG	-7.07 [b,f]	$K_{app}=2\times10^5$ M^{-1}	
KLA120	KLLAKAALKWLL<br>KALKAA		POPC<br>POPG	-4.95 [b,f]<br>-7.13 [b,f]	K_{app}=5500 M^{-1}<br>$K_{app}=2.2\times10^5$ M^{-1}	
KLA140	KALKKLLAKWLA<br>AAKALL		POPC<br>POPG	-5.14 [b,f]<br>-6.83 [b,f]	K_{app}=7500 M^{-1}<br>$K_{app}=1.3\times10^5$ M^{-1}	
KLA160	KLAAALLKKWKK<br>LAAALL		POPC<br>POPG	-5.14 [b,f]<br>-7.02 [b,f]	K_{app}=7500 M^{-1}<br>$K_{app}=1.8\times10^5$ M^{-1}	
KLA180	KALAALLKKWAK<br>LLAALK		POPG	-6.83 [b,f]	$K_{app}=1.3\times10^5$ M^{-1}	

Reference:

1. Wenk MR, Seelig J (1998) Magainin 2 amide interaction with lipid membranes: calorimetric detection of peptide binding and pore formation. Biochemistry 37: 3909-3916.
2. Wieprecht T, Beyermann M, Seelig J (1999) Binding of Antibacterial Magainin Peptides to Electrically Neutral Membranes: Thermodynamics and Structure. Biochemistry 38: 10377-10387.
3. Wieprecht T, Apostolov O, Seelig J (2000) Binding of the antibacterial peptide magainin 2 amide to small and large unilamellar vesicles. Biophysical Chemistry 85: 187-198.
4. Wieprecht T, Dathe M, Schumann M, Krause E, Beyermann M, et al. (1996) Conformational and Functional Study of Magainin 2 in Model Membrane Environments Using the New Approach of Systematic Double-d-Amino Acid Replacement. Biochemistry 35: 10844-10853.
5. Wieprecht T, Dathe M, Beyermann M, Krause E, Maloy WL, et al. (1997) Peptide Hydrophobicity Controls the Activity and Selectivity of Magainin 2 Amide in Interaction with Membranes. Biochemistry 36: 6124-6132.
6. Gregory S, Pokorny A, Almeida P (2009) Magainin 2 Revisited: A Test of the Quantitative Model for the All-or-None Permeabilization of Phospholipid Vesicles. Biophysical Journal 96: 116-131.
7. Schoch P, Sargent DF (1980) Quantitative analysis of the binding of melittin to planar lipid bilayers allowing for the discrete-charge effect. Biochimica et Biophysica Acta (BBA) - Biomembranes 602: 234-247.
8. Beschiaschvili G, Seelig J (1990) Melittin binding to mixed phosphatidylglycerol/phosphatidylcholine membranes. Biochemistry 29: 52-58.
9. Rex S, Schwarz G (1998) Quantitative studies on the melittin-induced leakage mechanism of lipid vesicles. Biochemistry 37: 2336-2345.
10. Allende D, Vidal A, Simon SA, McIntosh TJ (2003) Bilayer interfacial properties modulate the binding of amphipathic peptides. Chemistry and Physics of Lipids 122: 65-76.
11. Allende D, Simon SA, McIntosh TJ (2005) Melittin-Induced Bilayer Leakage Depends on Lipid Material Properties: Evidence for Toroidal Pores. Biophysical Journal 88: 1828-1837.
12. Fernández-Vidal M, White SH, Ladokhin AS (2011) Membrane Partitioning: “Classical” and “Nonclassical” Hydrophobic Effects. The Journal of Membrane Biology 239: 5-14.
13. Rapaport D, Shai Y (1991) Interaction of fluorescently labeled pardaxin and its analogues with lipid bilayers. Journal of Biological Chemistry 266: 23769-23775.
14. Bhunia A, Domadia PN, Torres J, Hallock KJ, Ramamoorthy A, et al. (2010) NMR structure of pardaxin, a pore-forming antimicrobial peptide, in lipopolysaccharide micelles: mechanism of outer membrane permeabilization. J Biol Chem 285: 3883-3895.
15. Pouny Y, Rapaport D, Mor A, Nicolas P, Shai Y (1992) Interaction of antimicrobial dermaseptin and its fluorescently labeled analogs with phospholipid membranes. Biochemistry 31: 12416-12423.
16. Gaidukov L, Fish A, Mor A (2003) Analysis of Membrane-Binding Properties of Dermaseptin Analogues: Relationships between Binding and Cytotoxicity. Biochemistry 42: 12866-12874.
17. Verly RM, Rodrigues MA, Daghastanli KRP, Denadai AML, Cuccovia IM, et al. (2008) Effect of cholesterol on the interaction of the amphibian antimicrobial peptide DD K with liposomes. Peptides 29: 15-24.
18. Gazit E, Boman A, Boman HG, Shai Y (1995) Interaction of the Mammalian Antibacterial Peptide Cecropin P1 with Phospholipid Vesicles. Biochemistry 34: 11479-11488.
19. Gregory SM, Cavenaugh A, Journigan V, Pokorny A, Almeida PFF (2008) A Quantitative Model for the All-or-None Permeabilization of Phospholipid Vesicles by the Antimicrobial Peptide Cecropin A. Biophysical Journal 94: 1667-1680.
20. Silvestro L, Gupta K, Weiser JN, Axelsen PH (1997) The concentration-dependent membrane activity of cecropin A. Biochemistry 36: 11452-11460.
21. Clark KS, Svetlovics J, McKeown AN, Huskins L, Almeida PF (2011) What Determines the Activity of Antimicrobial and Cytolytic Peptides in Model Membranes. Biochemistry 50: 7919-7932.

22. Almeida PF, Pokorny A (2009) Mechanisms of Antimicrobial, Cytolytic, and Cell-Penetrating Peptides: From Kinetics to Thermodynamics. Biochemistry 48: 8083-8093.
23. Lewis JR, Cafiso DS (1999) Correlation between the free energy of a channel-forming voltage-gated peptide and the spontaneous curvature of bilayer lipids. Biochemistry 38: 5932-5938.
24. Abraham T, Lewis RN, Hodges RS, McElhaney RN (2005) Isothermal titration calorimetry studies of the binding of a rationally designed analogue of the antimicrobial peptide gramicidin s to phospholipid bilayer membranes. Biochemistry 44: 2103-2112.
25. Bastos M, Bai G, Gomes P, Andreu D, Goormaghtigh E, et al. (2008) Energetics and Partition of Two Cecropin-Melittin Hybrid Peptides to Model Membranes of Different Composition. Biophysical Journal 94: 2128-2141.
26. Bhargava K, Feix JB (2004) Membrane Binding, Structure, and Localization of Cecropin-Mellitin Hybrid Peptides: A Site-Directed Spin-Labeling Study. Biophys J 86: 329-336.
27. Sood R, Domanov Y, Pietiäinen M, Kontinen VP, Kinnunen PKJ (2008) Binding of LL-37 to model biomembranes: Insight into target vs host cell recognition. Biochimica et Biophysica Acta (BBA) - Biomembranes 1778: 983-996.
28. Andrushchenko VV, Aarabi MH, Nguyen LT, Prenner EJ, Vogel HJ (2008) Thermodynamics of the interactions of tryptophan-rich cathelicidin antimicrobial peptides with model and natural membranes. Biochimica et Biophysica Acta (BBA) - Biomembranes 1778: 1004-1014.
29. Ladokhin AS, Selsted ME, White SH (1997) Bilayer Interactions of Indolicidin, a Small Antimicrobial Peptide Rich in Tryptophan, Proline, and Basic Amino Acids. Biophysical Journal 72: 794-805.
30. Yamamoto N, Tamura A (2010) Designed low amphipathic peptides with alpha-helical propensity exhibiting antimicrobial activity via a lipid domain formation mechanism. Peptides 31: 794-805.
31. Dathe M, Meyer J, Beyermann M, Maul B, Hoischen C, et al. (2002) General aspects of peptide selectivity towards lipid bilayers and cell membranes studied by variation of the structural parameters of amphipathic helical model peptides. Biochimica et Biophysica Acta (BBA) - Biomembranes 1558: 171-186.
