# Supplementary material for: Activity Determinants of Helical Antimicrobial Peptides: A Large-Scale Computational Study
Source: PLoS One. 2013 Jun 12;8(6):e66440. doi: 10.1371/journal.pone.0066440 (PMC3680375; doi:10.1371/journal.pone.0066440)
Supplement: Table S2 — Theoretical transfer energies and biological activities of the peptides. (PDF) [file pone.0066440.s003.pdf]

Table S2. Theoretical transfer energies and biological activities of the peptides.

Minimal inhibitory concentrations(MIC) have been converted into μM. The colony formation unit (CFU, cell/ml) is the bacterial concentration at which bactericidal activity is measured. Hemolytic activity is described as the percentage of erythrocyte (RBC represents Red Blood Cell, hRBC is from human) lysis after treatment of certain concentration of the peptide and certain time (30min-3hr).

A) Peptides from arthropods:

PDB	Name	10% Anionic	30% Anionic	MIC(μM)			CFU	Hemolytic	Ref.
				G-		G+			
				E. Coli	P.A.	S.Aureus			
1f0d	CA(1−8)−MA(1−12) *	-9.3±2.2	-15.6±0.3	3.125	1.56	3.125	2x10^6	0%at100 μM<br>4% hRBC	[1]
1f0f	CA(1−8)−MA(1−12), $G^9I^{10}G^{11}$ deletion*	-5.0±2.8	-9.5±1.5	6.25	3.125	3.125	2x10^6	0%at100 μM<br>4% hRBC	
1f0e	CA(1−8)−MA(1−12), $G^9I^{10}G^{11} \rightarrow P^*$	-5.5±0.5	-12.1±2.4	6.25	1.56	3.125	2x10^6	0%at100 μM<br>4% hRBC	
2jmy	CM15, CecropinA-Melittin hybrid	-10.5±0.9	-13.2±0.3	0.5-1	2-4	0.5-2	1x10^5	45%at64 μM<br>1% hRBC (1hr)	[2,3]
2mlt	Melittin	-16.4±0.6	-19.7±2.5	2,3.8	2	0.5	2x10^6	50%at1.7 μM<br>1x10^9<br>hRBC(1hr)	[4,5]
	Cecropin A▲	-15.5±1.4	-19.5±1.9	0.35	2.6	?	2x10^5	13%at 145 μM<br>Sheep RBC	[6,7]
1t51	IsCT	-8.5±1.1	-10.6±0.5	4	2	2	2x10^6	72%at50 μM<br>50%at18 μM<br>4% hRBC (1hr)<br>50%at70 μM<br>(sheep RBC 1hr)	[8]
1t52	IsCT E7K	-9.6±0.5	-11.0±1.5	2	2	2	2x10^6	30%at50 μM<br>4% hRBC	
1t54	IsCT W6A	-8.4±0.9	-9.9±0.5	64	32	64	2x10^6	5.0%at50 μM<br>4% hRBC	
1t55	IsCT E7K, G8P, S11K	-9.2±0.6	-14.0±1.1	2	2	2	2x10^6	0.0%at50 μM<br>4% hRBC	
2pco	Latarcin 1	-2.1±0.5	-11.5±1.9	0.7,1.0	4.1	?	1x10^5	20%at80 μM<br>2x10^7<br>Rabbit<br>RBC 3hr	[9,10]
2g9p	Latarcin 2a	-11.1±1.4	-17.2±1.6	0.5,0.7	6.7	?	10^5	50%at6 μM<br>20%at5 μM<br>hRBC	[11,12]
1zrw	Spinigerin	-9.8±1.8	-9.6±2.2	16	16	8	(2-4)x10^6	0%at100 μM<br>4% hRBC	[13]
1zrx	Stomoxyn	-7.8±0.4	-12.1±0.7	0.19-0.39	0.39-0.78	?	?	8%at100 μM<br>4% hRBC	[14]
2l3i	Oxyopinin 4a	-11.1±0.7	-17.1±0.6	0.5		10	?prev	50%at 7 μM	[15]
1kv4	Moricin	-16.7±1.4	-21.3±3.3	0.31	0.81	0.21	?	?	[16]
2jr8	Manduca Sexta Moricin	-10.8±0.7	-19.2±0.6	1.38		1.38-2.76	5×10^5		[17]
2k38	Cupiennin 1a	-11.0±0.5	-16.3±1.1	0.31-0.63	0.31-0.63	0.31-0.63	(1.7-3.8)x10^5	50%at24.4 μM	[18]
1d7n	Mastoparan M	-10.5±1.1	-12.0±1.6	4.22	4.22	2.11	10^5	10% at50 μM<br>1% sheep RBC<br>1hr	[19,20]
2czp	Mastoparan X	-5.9±0.8	-8.5±0.2					Visible at 9.6μM	[21]

* CecropinA-Magainin hybrid ▲ Built as ideal alpha-helix **?** No data available NE No Effect.

B) Peptides from amphibian skin secretions:

PDB	Name	10% Anionic	30% Anionic	MIC(μM)			CFU	Hemolytic	Ref.
				G-		G+			
				E. Coli	P.A.	S.Aureus			
1vm2	mutant peptide A2	-8.2±0.1	-9.4±0.2	250	?	?	$1x10^6$	?	[22]
1vm3	mutant peptide A3	-8.8±0.4	-9.4±0.1	NE	?	?	$1x10^6$	?	
1vm4	mutant peptide A4	-8.7±0.5	-10.3±0.1	100	?	?	$1x10^6$	?	
1vm5	Aurein 1.2	-9.1±0.8	-10.7±0.1	75	?	8	$1x10^6$	?	
1o53	Nontoxic membrane anchor E. coli enzyme IIA(Glucose)	-5.3±0.7	-7.4±0.9	NE	?	?	$1x10^6$	?	
2f3a	LLAA(LL-37 derived Aurein 1.2 analog)	-9.6±0.8	-13.0±0.3	20	?	?	$1.8x10^6$	?	[23]
2jpy	Phylloseptin-H2	-13.9±0.5	-14.9±0.3	3.7	7.6	1.9	$1x10^5$	2.05%at64 μM hRBC(30min)	[24,25]
2jq0	Phylloseptin-H1	-10.3±0.2	-11.9±0.2	1.9	7.9	3.9	$1x10^5$	0.98%at32 μM hRBC(30min)	
2jq1	Phylloseptin-H3	-10.6±0.9	-11.7±0.1	4.1	8.2	4.1	$1x10^5$	?	
2g9l	Gaegurin -4	-14.7±0.9	-17.8±0.8	20.00	26.67		$1x10^6$	2%at100 μM 10% hRBC	[26-28]
2k10	Ranatuerin-2CSa	-15.4±1.3	-18.2±0.5	5	?	10	$1x10^6$	50%at150 μM $1x10^7$ hRBC(1hr)	[29,30]
2k9b	Dermadistinctin K	-5.0±2.2	-8.8±2.3	?	?	?	?	35%at46.7 μM Peritoneal cell(4hr)	[31]
	Dermaseptin-S1▲	-7.7±0.1	-11.2±1.2	12	>24	6	$5x10^5$	50%at>100μM 10^9 hRBC (30min) 50%at 12 μM hRBC(3hr)	[32]
1xkm	Distinctin	-6.6±1.6	-9.5±0.5	14.5	28,29	28,29	$1x10^4$	No hemolysis upto 1mM	[33,34]
2mag	Magainin 2	-7.6±0.6	-10.7±1.0	38+	76		$(1-5)x10^5$	50%at430 μM $1.8x10^8$ hRBC(30min)	[35,36]

▲ Built as ideal alpha-helix + Inconsistent values reported by different articles.

C) Peptides from cathelicidin family:

PDB	Name	10% Anionic	30% Anionic	MIC(μM)			CFU	Hemolytic	Ref.
				G-		G+			
				E. Coli	P.A.	S.Aureus			
1fry	SMAP-29(sheep cathelicidin)	-10.5±3.3	-17.0±0.6	0.25	1.25	0.5	(1.0-2.0)x10^5	19.4%at20 μM 10%hRBC	[37]
1hu5	Ovispirin-1	-12.1±1.2	-16.5±1.1	?	1.66	1	4x10^5	70.2% at 35 μM 5% hRBC 50% at 13.5 μM 3.5% hRBC 30min	[38-41]
1hu6	Novispirin G10	-7.6±2.5	-13.0±0.2	3.5	2.97	4.6	4x10^5	2.50%at 36 μM 5% hRBC 50% at 1470 μM	
1hu7	Novispirin T7	-6.3±2.6	-11.4±0.4	?	4.64	3.3	4x10^5	10%at35 μM 5% hRBC	
2k6o	LL-37(human cathelicidin)$^+$	-15.7±0.5	-19.1±1.9	27.8	27.8	55.6	1x10^5	50%at~75 μM 5% hRBC(1hr)	[42,43]
2lmf	LL-27(LL-37 N terminus 1-27)	-5.1±2.0	-8.6±0.4	160		24			[44]
2f3a	LLAA(LL37 derived Aurein1.2 analog)	-9.6±0.8	-13.0±0.3	20	?	?	1.8x10^6	?	[23]
2fbs	FK-13(LL37 core peptide)	-6.7±0.9	-10.1±1.0	40	?	?	2x10^6	?	[45]
1lyp	CAP18 (rabbit)	-14.7±1.6	-25.2±1.5	2.5	5.0	>5.0(~20 ?)	5-10x10^3	0% upto 500 μM	[46,47]
2amn	Fowlicidin-1(chicken cathelicidin)	-8.8±1.0	-17.4±0.6	4.0	?	0.5	5x10^5	50%at6 μM 0.5% hRBC 2hr	[48]
2gdl	Fowlicidin-2(chicken cathelicidin)	-6.3±3.0	-10.1±3.8	4-8	?	1	4x10^5	50%at100 μM hRBC	[49]
2hfr	Fowlicidin-3(chicken cathelicidin)	-12.6±2.0	-13.6±1.6	2	?	1	4x10^5	50%at9 μM hRBC	[49]

D) Miscellaneous peptides :

PDB	Name	10% Anionic	30% Anionic	MIC(µM)			CFU	Hemolytic	Ref.
				G-		G+			
				E. Coli	P.A.	S.Aureus			
1z64	Pleurocidin[+]	-8.2±0.6	-10.8±0.7	2.75	35	26.35	$2x10^{7}$	29%at50 µM 4% hRBC	[52-54]
2jos	Piscidin 1	-11.6±0.7	-13.4±1.0	3.1	12.5	3.1	$1x10^{5}$	50%at12 µM 5% hRBC 30min	[55,56]
	Warnericin-RK▲	-12.7±0.9	-13.3±0.3	20.0	20.0	20.0	$1x10^{6}$	50%at1.2 µM 1% hRBC 30min	[57]
2kam	δ-hemolysin	-13.0±0.6	-14.1±0.4	NA	NA	NA	NA	50% at8-10 µM(guinea pig RBC 30min)	[57,58]
1p0g	HP(2-20)	-7.6±0.8	-11.0±0.4	3.13,12.5	6.25	12.5	?	50% at ~150 µM (low salt buffer) 15% at 215 µM (high salt buffer)	[59,60]
1p0j	HP(2-20) Analog1	-8.6±0.4	-13.2±0.6	0.78,6.25	3.13	3.13			
1p0l	HP(2-20) Analog2	-7.4±0.6	-11.9±0.2	3.13,6.25	6.25	6.25			
1p0o	HP(2-20) Analog3	-8.2±1.3	-12.4±0.3	1.56,3.13	3.13	1.56,3.13		0% at 100uM 8% hRBC	
1amt	Alamethicin	-8.2±0.2	-8.0±0.2	7.64	?	?	?	50%at 30µM $1.8x10^{8}$cell/ml hRBC(30min)	[61,62]
1xc0	Pardaxin	-20.3±2.0	-22.7±2.8	13	25	?	$1x10^{6}$	50%at 50 µM 5% hRBC 30min	[63,64]

▲ Built from ideal alpha-helix

[+] Inconsistent results between different articles.

NA No activity against the target organism.

Reference:

1. Shin SY, Kang JH, Jang SY, Kim Y, Kim KL, et al. (2000) Effects of the hinge region of cecropin A(1-8)-magainin 2(1-12), a synthetic antimicrobial peptide, on liposomes, bacterial and tumor cells. Biochimica et Biophysica Acta (BBA) - Biomembranes 1463: 209-218.
2. Sato H, Feix JB (2006) Osmoprotection of bacterial cells from toxicity caused by antimicrobial hybrid peptide CM15. Biochemistry 45: 9997-10007.
3. Sato H, Feix JB (2008) Lysine-Enriched Cecropin-Mellitin Antimicrobial Peptides with Enhanced Selectivity. Antimicrobial Agents and Chemotherapy 52: 4463-4465.
4. Zhu WL, Nan YH, Hahm K-S, Shin SY (2007) Cell selectivity of an antimicrobial peptide melittin diastereomer with D-amino acid in the leucine zipper sequence. Journal of Biochemistry and Molecular Biology 40: 1090-1094.
5. Pandey BK, Ahmad A, Asthana N, Azmi S, Srivastava RM, et al. (2010) Cell-Selective Lysis by Novel Analogues of Melittin against Human Red Blood Cells and Escherichia coli. Biochemistry 49: 7920-7929.
6. Andreu D, Merrifield RB, Steiner H, Boman HG (1985) N-Terminal analogs of cecropin A: synthesis, antibacterial activity, and conformational properties. Biochemistry 24: 1683-1688.
7. Steiner H, Andreu D, Merrifield RB (1988) Binding and action of cecropin and cecropin analogues: Antibacterial peptides from insects. Biochimica et Biophysica Acta (BBA) - Biomembranes 939: 260-266.
8. Lee K, Shin SY, Kim K, Lim SS, Hahm K-S, et al. (2004) Antibiotic activity and structural analysis of the scorpion-derived antimicrobial peptide IsCT and its analogs. Biochemical and Biophysical Research Communications 323: 712-719.
9. Kozlov SA, Vassilevski AA, Feofanov AV, Surovoy AY, Karpunin DV, et al. (2006) Latarcins, Antimicrobial and Cytolytic Peptides from the Venom of the Spider Lachesana tarabaevi (Zodariidae) That Exemplify Biomolecular Diversity. Journal of Biological Chemistry 281: 20983-20992.
10. Vasilevskiĭ AA, Kozlov SA, Zhmak MN, Kudelina IA, Dubovskiĭ PV, et al. (2007) Synthetic analogues of antimicrobial peptides from the venom of the Central Asian spider Lachesana tarabaevi. Bioorganicheskaia Khimiia 33: 405-412.
11. Shlyapnikov YM, Andreev YA, Kozlov SA, Vassilevski AA, Grishin EV (2008) Bacterial production of latarcin 2a, a potent antimicrobial peptide from spider venom. Protein Expression and Purification 60: 89-95.
12. Grishin EV, Polyansky AA, Vassilevski AA, Volynsky PE, Vorontsova OV, et al. (2009) N-terminal amphipathic helix as a trigger of hemolytic activity in antimicrobial peptides: A case study in latarcins. FEBS Letters 583: 2425-2428.
13. Lee KH, Shin SY, Hong JE, Yang S-T, Kim JI, et al. (2003) Solution structure of termite-derived antimicrobial peptide, spinigerin, as determined in SDS micelle by NMR spectroscopy. Biochemical and Biophysical Research Communications 309: 591-597.
14. Boulanger N, Munks RJL, Hamilton JV, Vovelle F, Brun R, et al. (2002) Epithelial Innate Immunity: A NOVEL ANTIMICROBIAL PEPTIDE WITH ANTIPARASITIC ACTIVITY IN THE BLOOD-SUCKING INSECT STOMOXYS CALCITRANS. Journal of Biological Chemistry 277: 49921-49926.
15. Dubovskii PV, Vassilevski AA, Samsonova OV, Egorova NS, Kozlov SA, et al. (2011) Novel lynx spider toxin shares common molecular architecture with defense peptides from frog skin. FEBS Journal 278: 4382-4393.
16. Satoe K, Ai A, Mitsuhiro M, Jun I, Minoru Y (2005) Interactions of an Antimicrobial Peptide Moricin with Lipid Bilayers. Pept Sci 2004: 251-254.
17. Dai H, Rayaprolu S, Gong Y, Huang R, Prakash O, et al. (2008) Solution structure, antibacterial activity, and expression profile of Manduca sexta moricin. Journal of Peptide Science 14: 855-863.

18. Kuhn-Nentwig L, Müller J, Schaller J, Walz A, Dathe M, et al. (2002) Cupiennin 1, a New Family of Highly Basic Antimicrobial Peptides in the Venom of the Spider Cupiennius salei(Ctenidae). Journal of Biological Chemistry 277: 11208-11216.
19. Li M-L, Liao R-W, Qiu J-W, Wang Z-J, Wu T-M (2000) Antimicrobial activity of synthetic all-mastoparan M. International Journal of Antimicrobial Agents 13: 203-208.
20. Murata K, Shinada T, Ohfune Y, Hisada M, Yasuda A, et al. (2008) Novel mastoparan and protonectin analogs isolated from a solitary wasp, Orancistrocerus drewseni drewseni. Amino Acids 37: 389-394.
21. Hirai Y, Kuwada M, Yasuhara T, Yoshida H, Nakajima T (1979) A new mast cell degranulating peptide homologous to mastoparan in the venom of Japanese hornet (Vespa xanthoptera). Chemical & Pharmaceutical Bulletin 27: 1945-1946.
22. Wang G, Li Y, Li X (2005) Correlation of Three-dimensional Structures with the Antibacterial Activity of a Group of Peptides Designed Based on a Nontoxic Bacterial Membrane Anchor. Journal of Biological Chemistry 280: 5803-5811.
23. Li X, Li Y, Peterkofsky A, Wang G (2006) NMR studies of aurein 1.2 analogs. Biochimica Et Biophysica Acta 1758: 1203-1214.
24. Bloch, Jr., Leite JRSA, Silva LP, Rodrigues MIS, Prates MV, et al. (2005) Phylloseptins: a novel class of anti-bacterial and anti-protozoan peptides from the Phyllomedusa genus. Peptides 26: 565-573.
25. Bechinger B, Resende JM, Moraes CM, Prates MV, Cesar A, et al. (2008) Solution NMR structures of the antimicrobial peptides phylloseptin-1, -2, and -3 and biological activity: the role of charges and hydrogen bonding interactions in stabilizing helix conformations. Peptides 29: 1633-1644.
26. Park JM, Jung JE, Lee BJ (1994) Antimicrobial Peptides from the Skin of a Korean Frog, Rana rugosa. Biochemical and Biophysical Research Communications 205: 948-954.
27. Won HS, Park SH, Kim HE, Hyun B, Kim M, et al. (2002) Effects of a tryptophanyl substitution on the structure and antimicrobial activity of C‐terminally truncated gaegurin 4. European Journal of Biochemistry 269: 4367-4374.
28. Chi S-W, Kim J-S, Kim D-H, Lee S-H, Park Y-H, et al. (2007) Solution structure and membrane interaction mode of an antimicrobial peptide gaegurin 4. Biochemical and Biophysical Research Communications 352: 592-597.
29. Conlon JM, al-Dhaheri A, al-Mutawa E, al-Kharrge R, Ahmed E, et al. (2007) Peptide defenses of the Cascades frog Rana cascadae: implications for the evolutionary history of frogs of the Amerana species group. Peptides 28: 1268-1274.
30. Subasinghage AP, Conlon JM, Hewage CM (2008) Conformational analysis of the broad-spectrum antibacterial peptide, ranatuerin-2CSa: Identification of a full length helix-turn-helix motif. Biochimica et Biophysica Acta (BBA) - Proteins & Proteomics 1784: 924-929.
31. Brand GD, Leite JRSA, Silva LP, Albuquerque S, Prates MV, et al. (2002) Dermaseptins from Phyllomedusa oreades andPhyllomedusa distincta. Journal of Biological Chemistry 277: 49332-49340.
32. Savoia D, Guerrini R, Marzola E, Salvadori S (2008) Synthesis and antimicrobial activity of dermaseptin S1 analogues. Bioorganic & Medicinal Chemistry 16: 8205-8209.
33. Batista CVF, Scaloni A, Rigden DJ, Silva LR, Rodrigues Romero A, et al. (2001) A novel heterodimeric antimicrobial peptide from the tree-frog Phyllomedusa distincta. FEBS Letters 494: 85-89.
34. Raimondo D, Andreotti G, Saint N, Amodeo P, Renzone G, et al. (2005) A folding-dependent mechanism of antimicrobial peptide resistance to degradation unveiled by solution structure of distinctin. Proceedings of the National Academy of Sciences of the United States of America 102: 6309-6314.
35. Wieprecht T, Dathe M, Beyermann M, Krause E, Maloy WL, et al. (1997) Peptide Hydrophobicity Controls the Activity and Selectivity of Magainin 2 Amide in Interaction with Membranes. Biochemistry 36: 6124-6132.

36. Zasloff M, Martin B, Chen HC (1988) Antimicrobial activity of synthetic magainin peptides and several analogues. Proceedings of the National Academy of Sciences of the United States of America 85: 910-913.
37. Skerlavaj B, Benincasa M, Risso A, Zanetti M, Gennaro R (1999) SMAP-29: a potent antibacterial and antifungal peptide from sheep leukocytes. FEBS Letters 463: 58-62.
38. Sawai MV, Waring AJ, Kearney WR, McCray PB, Forsyth WR, et al. (2002) Impact of single-residue mutations on the structure and function of ovispirin/novispirin antimicrobial peptides. Protein Engineering 15: 225-232.
39. Steinstraesser L, Tack BF, Waring AJ, Hong T, Boo LM, et al. (2002) Activity of Novispirin G10 against Pseudomonas aeruginosa In Vitro and in Infected Burns. Antimicrobial Agents and Chemotherapy 46: 1837-1844.
40. Jacobsen F, Mohammadi-Tabrisi A, Hirsch T, Mittler D, Mygind PH, et al. (2007) Antimicrobial activity of the recombinant designer host defence peptide P-novispirin G10 in infected full-thickness wounds of porcine skin. Journal of Antimicrobial Chemotherapy 59: 493-498.
41. Eckert R, Qi F, Yarbrough DK, He J, Anderson MH, et al. (2006) Adding Selectivity to Antimicrobial Peptides: Rational Design of a Multidomain Peptide against Pseudomonas spp. Antimicrobial Agents and Chemotherapy 50: 1480-1488.
42. Bals R, Wang X, Zasloff M, Wilson JM (1998) The peptide antibiotic LL-37/hCAP-18 is expressed in epithelia of the human lung where it has broad antimicrobial activity at the airway surface. Proceedings of the National Academy of Sciences of the United States of America 95: 9541-9546.
43. Oren Z, Lerman JC, Gudmundsson GH, Agerberth B, Shai Y (1999) Structure and organization of the human antimicrobial peptide LL-37 in phospholipid membranes: relevance to the molecular basis for its non-cell-selective activity. Biochemical Journal 341: 501-513.
44. Wang G, Elliott M, Cogen AL, Ezell EL, Gallo RL, et al. (2012) Structure, Dynamics, and Antimicrobial and Immune Modulatory Activities of Human LL-23 and Its Single-Residue Variants Mutated on the Basis of Homologous Primate Cathelicidins. Biochemistry 51: 653-664.
45. Li X, Li Y, Han H, Miller DW, Wang G (2006) Solution Structures of Human LL-37 Fragments and NMR-Based Identification of a Minimal Membrane-Targeting Antimicrobial and Anticancer Region. Journal of the American Chemical Society 128: 5776-5785.
46. Travis SM, Anderson NN, Forsyth WR, Espiritu C, Conway BD, et al. (2000) Bactericidal Activity of Mammalian Cathelicidin-Derived Peptides. Infection and Immunity 68: 2748-2755.
47. Larrick JW, Hirata M, Shimomoura Y, Yoshida M, Zheng H, et al. (1993) Antimicrobial activity of rabbit CAP18-derived peptides. Antimicrobial Agents and Chemotherapy 37: 2534-2539.
48. Xiao Y, Dai H, Bommineni YR, Soulages JL, Gong Y-X, et al. (2006) Structure–activity relationships of fowlicidin-1, a cathelicidin antimicrobial peptide in chicken. FEBS Journal 273: 2581-2593.
49. Xiao Y, Herrera AI, Bommineni YR, Soulages JL, Prakash O, et al. (2009) The Central Kink Region of Fowlicidin-2, an α-Helical Host Defense Peptide, Is Critically Involved in Bacterial Killing and Endotoxin Neutralization. Journal of Innate Immunity 1: 268-280.
50. Li Q, Dong C, Deng A, Katsumata M, Nakadai A, et al. (2005) Hemolysis of Erythrocytes by Granulysin-Derived Peptides but Not by Granulysin. Antimicrob Agents Chemother 49: 388-397.
51. Lai Y-P, Peng Y-F, Zuo Y, Li J, Huang J, et al. (2005) Functional and structural characterization of recombinant dermcidin-1L, a human antimicrobial peptide. Biochemical and Biophysical Research Communications 328: 243-250.
52. Cole AM, Weis P, Diamond G (1997) Isolation and Characterization of Pleurocidin, an Antimicrobial Peptide in the Skin Secretions of Winter Flounder. Journal of Biological Chemistry 272: 12008-12013.
53. Yoshida K, Mukai Y, Niidome T, Takashi C, Tokunaga Y, et al. (2001) Interaction of pleurocidin and its analogs with phospholipid membrane and their antibacterial activity. Journal of Peptide Research 57: 119-119.
54. Lee J, Lee DG (2008) Structure-antimicrobial activity relationship between pleurocidin and its enantiomer. Experimental & Molecular Medicine 40: 370-376.

55. Silphaduang U, Noga EJ (2001) Antimicrobials: Peptide antibiotics in mast cells of fish. Nature 414: 268-269.
56. Chekmenev EY, Vollmar BS, Forseth KT, Manion MN, Jones SM, et al. (2006) Investigating molecular recognition and biological function at interfaces using piscidins, antimicrobial peptides from fish. Biochimica Et Biophysica Acta 1758: 1359-1372.
57. Verdon J, Berjeaud J-M, Lacombe C, Héchard Y (2008) Characterization of anti-Legionella activity of warnericin RK and delta-lysin I from Staphylococcus warneri. Peptides 29: 978-984.
58. Dhople VM, Nagaraj R (2005) Conformation and activity of δ-lysin and its analogs. Peptides 26: 217-225.
59. Ribeiro P, x, cia D, Medina-Acosta E (2003) Prevention of lethal murine candidiasis using HP (2–20), an antimicrobial peptide derived from the N-terminus of Helicobacter pylori ribosomal protein L1. Peptides 24: 1807-1814.
60. Lee Kwang H, Lee Dong G, Park Y, Kang D-I, Shin Song Y, et al. (2006) Interactions between the plasma membrane and the antimicrobial peptide HP (2-20) and its analogues derived from Helicobacter pylori. Biochemical Journal 394: 105-105.
61. Dathe M, Kaduk C, Tachikawa E, Melzig MF, Wenschuh H, et al. (1998) Proline at position 14 of alamethicin is essential for hemolytic activity, catecholamine secretion from chromaffin cells and enhanced metabolic activity in endothelial cells. Biochimica et Biophysica Acta (BBA) - Biomembranes 1370: 175-183.
62. Wenschuh H, Beyermann M, Haber H, Seydel JK, Krause E, et al. (1995) Stepwise Automated Solid Phase Synthesis of Naturally Occurring Peptaibols Using FMOC Amino Acid Fluorides. J Org Chem 60: 405-410.
63. Rapaport D, Shai Y (1991) Interaction of fluorescently labeled pardaxin and its analogues with lipid bilayers. Journal of Biological Chemistry 266: 23769-23775.
64. Oren Z, Shai Y (1996) A Class of Highly Potent Antibacterial Peptides Derived from Pardaxin, A Pore‐Forming Peptide Isolated from Moses Sole Fish Pardachirus marmoratus. European Journal of Biochemistry 237: 303-310.
