# Supplementary material for: Activity Determinants of Helical Antimicrobial Peptides: A Large-Scale Computational Study
Source: PLoS One. 2013 Jun 12;8(6):e66440. doi: 10.1371/journal.pone.0066440 (PMC3680375; doi:10.1371/journal.pone.0066440)
Supplement: Table S3 — Biological activity of the peptides that do not have PDB structures. (PDF) [file pone.0066440.s004.pdf]

Table S3. Biological activity of the peptides that do not have PDB structures.

Peptide	MIC μM<br>E. Coli	EC50 μM	Ref.
PGLa	4.06-8.12	406	[1]
Gramicidin S 14dK4	3.1-6.2		[2]
Dermaseptin S1		12	[3]
Dermaseptin S4		1.4 ± 0.2	
K4K20-S4	1-16	0.5 ± 0.1	[3,4]
K4-S4(1-16)a	1-8	10 ±0.5	
K4-S4(1-13)a	2-16	57 ± 3	
K4-S4(1-10)a		>100	
Cecropin P	3.3		[5]
DL-1	3.8	4	[6]
LAP1	24		[7]
LAP2	16		
LAP3	24		
LAP4	55		
LAP5	6.0		
LAP6	6.0		
KLA80	3	55	[8]
KLA100	3.1	55	
KLA120	6.1	15	
KLA140	6.0	45	
KLA160	5.9	60	
KLA180	5.9	40	

References:

1. Wieprecht T, Beyermann M, Seelig J (1999) Binding of Antibacterial Magainin Peptides to Electrically Neutral Membranes: Thermodynamics and Structure. Biochemistry 38: 10377-10387.
2. Kondejewski LH, Jelokhani-Niaraki M, Farmer SW, Lix B, Kay CM, et al. (1999) Dissociation of Antimicrobial and Hemolytic Activities in Cyclic Peptide Diastereomers by Systematic Alterations in Amphipathicity. Journal of Biological Chemistry 274: 13181-13192.
3. Gaidukov L, Fish A, Mor A (2003) Analysis of Membrane-Binding Properties of Dermaseptin Analogues: Relationships between Binding and Cytotoxicity. Biochemistry 42: 12866-12874.
4. Navon-Venezia S, Feder R, Gaidukov L, Carmeli Y, Mor A (2002) Antibacterial Properties of Dermaseptin S4 Derivatives with In Vivo Activity. Antimicrobial Agents and Chemotherapy 46: 689-694.
5. Gazit E, Boman A, Boman HG, Shai Y (1995) Interaction of the Mammalian Antibacterial Peptide Cecropin P1 with Phospholipid Vesicles. Biochemistry 34: 11479-11488.
6. Dhople VM, Nagaraj R (2005) Conformation and activity of δ-lysin and its analogs. Peptides 26: 217-225.
7. Yamamoto N, Tamura A (2010) Designed low amphipathic peptides with α-helical propensity exhibiting antimicrobial activity via a lipid domain formation mechanism. Peptides 31: 794-805.
8. Dathe M, Meyer J, Beyermann M, Maul B, Hoischen C, et al. (2002) General aspects of peptide selectivity towards lipid bilayers and cell membranes studied by variation of the structural parameters of amphipathic helical model peptides. Biochimica et Biophysica Acta (BBA) - Biomembranes 1558: 171-186.
