# Supplementary material for: Activity Determinants of Helical Antimicrobial Peptides: A Large-Scale Computational Study
Source: PLoS One. 2013 Jun 12;8(6):e66440. doi: 10.1371/journal.pone.0066440 (PMC3680375; doi:10.1371/journal.pone.0066440)
Supplement: Table S4 — Comparison of the free energy from explicit simulations with experimental binding free energy and transfer energy <ΔW> from IMM1. (PDF) [file pone.0066440.s005.pdf]

Table S4. Comparison of the free energy from explicit simulations with experimental binding free energy and transfer energy <ΔW> from IMM1.

Peptide	Membrane	$\Delta G_{c,sim}^{0}$	ΔG_{c}^{0}	< ΔW>	Ref.
Lactoferricin	DOPC	-1.05±0.39		-0.9 ±0.1	[1]
	DOPG	-5.4±1.3	-7.38+	-7.9±0.7	[2,3]
Protegrin	DMPC/DMPG(7:3)	-2.4±0.8	-7*	-5.3 ±1.1	[4,5]
Indolicidin	DOPC	-0.05±0.46	-7.25	-1.1±1.2	[6,7]
	DOPC/DOPG(7:3)	-1.94±0.56	-8.97	-5.5±0.8	
Melittin	DOPC	-10.66 ±1.35	-5.4	-13.1±0.7	[8,9]

+ The experimental value is obtained for a truncated lactoferricin which has less charge, so the actual value should be more negative.

* The standard state of the experiment is assumed to be ΔG_{c}^{0}.

Reference:

1. Vivcharuk V, Tomberli B, Tolokh IS, Gray CG (2008) Prediction of binding free energy for adsorption of antimicrobial peptide lactoferricin B on a POPC membrane. Physical Review E 77.
2. Tolokh IS, Vivcharuk V, Tomberli B, Gray CG (2009) Binding free energy and counterion release for adsorption of the antimicrobial peptide lactoferricin B on a POPG membrane. Physical Review E 80.
3. Jing WG, Svendsen JS, Vogel HJ (2006) Comparison of NMR structures and model-membrane interactions of 15-residue antimicrobial peptides derived from bovine lactoferricin. Biochemistry and Cell Biology-Biochimie Et Biologie Cellulaire 84: 312-326.
4. Vivcharuk V, Kaznessis Y (2010) Free Energy Profile of the Interaction between a Monomer or a Dimer of Protegrin-1 in a Specific Binding Orientation and a Model Lipid Bilayer. The Journal of Physical Chemistry B 114: 2790-2797.
5. Lai JR, Epand RF, Weisblum B, Epand RM, Gellman SH (2006) Roles of salt and conformation in the biological and physicochemical behavior of protegrin-1 and designed analogues: Correlation of antimicrobial, hemolytic, and lipid bilayer-perturbing activities. Biochemistry 45: 15718-15730.
6. Yeh I-C, Ripoll DR, Wallqvist A (2012) Free Energy Difference in Indolicidin Attraction to Eukaryotic and Prokaryotic Model Cell Membranes. The Journal of Physical Chemistry B 116: 3387-3396.
7. Andrushchenko VV, Aarabi MH, Nguyen LT, Prenner EJ, Vogel HJ (2008) Thermodynamics of the interactions of tryptophan-rich cathelicidin antimicrobial peptides with model and natural membranes. Biochimica et Biophysica Acta (BBA) - Biomembranes 1778: 1004-1014.
8. Irudayam SJ, Berkowitz ML (2012) Binding and reorientation of melittin in a POPC bilayer: Computer simulations. Biochimica et Biophysica Acta (BBA) - Biomembranes 1818: 2975-2981.
9. Allende D, Simon SA, McIntosh TJ (2005) Melittin-Induced Bilayer Leakage Depends on Lipid Material Properties: Evidence for Toroidal Pores. Biophysical Journal 88: 1828-1837.
