# Supplementary material for: Activity Determinants of Helical Antimicrobial Peptides: A Large-Scale Computational Study
Source: PLoS One. 2013 Jun 12;8(6):e66440. doi: 10.1371/journal.pone.0066440 (PMC3680375; doi:10.1371/journal.pone.0066440)
Supplement: Table S5 — List of peptide mechanism from literature. (PDF) [file pone.0066440.s006.pdf]

Table S5. List of peptide mechanism from literature.
PDB id, binding energy and activity of these peptides can be found in the table S2.

Peptide	Mechanism
CA-MA hybrid peptides	Possibly pore forming because of voltage induced conductance[1]. But membrane broke down after certain amount of time.
CM15	Osmoprotection reveals that the pore size in bacterial membranes is 2.2-3.8nm in diameters[2].
Melittin	Pore forming in zwitterionic membrane (DTPC bilayer, R_{in}=21Å, R_{out}=38.5Å) [3]but detergent-like in anionic membranes[4,5].
Cecropin A	Possibly ion channel because voltage-dependent single channel conductance is recorded[6].
Latarcins	Latarcin1 or Latarcin7 seems to form pore in DOPE/DOPG and DPhPC[7]. But the rest latarcins may adopt detergent-like mechanism[8]. Evidences are not solid though. Latarcin2a is pore-forming in erythrocyte membrane suggested by osmoprotection[9].
Oxyopinins	Oxt1 and 2a were suggested to form pore on PC vesicles but not on PA/PE vesicles[10]. Oxt4a has a Rana-box at the terminus which resembles the structure of C terminus of gaegurin-4 and ranatuerin-2CSa[11]. Pore mechanism is speculated.
Cupiennin 1a	Toroidal pore mechanism is suggested in anionic lipids[12] to explain the mobility of head groups but may also attack intra-cellular target(inhibits nitric oxide synthase)[13]. However, the effect of peptide on DMPC is different from the anionic lipids.
Mastoparan M	No information but Mastoparan-X forms pore[14].
Aurein 1.2	Surface active instead of pore formation[15]
Gaegurin -4	Pore forming indicated by the ion permeability experiment[16].
Ranatuerin-2CSa	No information available but the peptide has a structure quite similar to Gaegurin-4
Dermadistinctin K	Possibly toroidal pore[17]. ^{31}P NMR line shape can only be explained if 50% head groups are randomly aligned and 50% head groups are oriented 2-30°
Dermaseptin-S1	Suggested to be detergent-like because it only induces leakage at extremely high peptide/lipid ratio[18].
Magainin 2	Form toroidal pore in both neutral(DMPC) and anionic membranes[19]. It may adopt detergent-like mechanism in eukaryotic cell membranes though[20].
SMAP-29	Possibly pore forming[21]. But no actual evidence was given.
Ovispirin	It orient predominately parallel to the membrane surface, thus is considered to be adopting carpet mechanism[22].
LL-37	Previously suggested to be 'carpet' mechanism because it orients parallel to membrane surface[23]. The pore structure in DOPC/DOPE bilayers is however determined from neutron diffraction[24]. The radius of the pore is 23-33Å.
CAP18	Formation of transient lesions under voltage[25]. Possibly pore forming.
Fowlicidin	Fowl-1 aggregates in membrane mimicking solvents [26]. But no actual evidence for pore formation.

(continued in next page)

(continued from previous page)

Peptide	Mechanism
Indolicidin	Tryptophan location indicates 'Carpet' model on PC membranes[27] but with increased fraction of PG in the membrane, indolicidin starts to order the lipid acyl chains and this may be an indication of pore formation[28].
Human granulysin	Possibly intra-cellular pathways[29] because cell died in apoptotic but not necrotic ways.
Pleurocidin	Indicated by dye release experiment, it forms pore like magainin[30].
Piscidin 1	Single channel experiments indicate toroidal pore[31].
Warnericin-RK	Osmotic protection show that the pore in erythrocyte membranes can be as large as 5.7nm in diameter[32]. The author suggested that pore formation is not likely to be stable.
δ-hemolysin	Crystal structure shows that the pore is formed by 6-8 peptides[33].
HP(2-20)	Generates size dependent dype release in osmoprotection experiment for both PC and PG vesicles[34]. Supporting pore forming mechanism.
Alamethicin	Barrel-stave pore in DLPC membrane[35].
Pardaxin	Voltage induced multi-level channel in neutral membrane[36]. However, it is suggested to adopt detergent-like mechanism in the presence of PG[37].

Reference:

1. Oh D, Shin SY, Lee S, Kang JH, Kim SD, et al. (2000) Role of the Hinge Region and the Tryptophan Residue in the Synthetic Antimicrobial Peptides, Cecropin A(1−8)−Magainin 2(1−12) and Its Analogues, on Their Antibiotic Activities and Structures. Biochemistry 39: 11855-11864.
2. Sato H, Feix JB (2006) Osmoprotection of bacterial cells from toxicity caused by antimicrobial hybrid peptide CM15. Biochemistry 45: 9997-10007.
3. Yang L, Harroun TA, Weiss TM, Ding L, Huang HW (2001) Barrel-Stave Model or Toroidal Model? A Case Study on Melittin Pores. Biophysical Journal 81: 1475-1485.
4. Bechinger B, Lohner K (2006) Detergent-like actions of linear amphipathic cationic antimicrobial peptides. Biochimica et Biophysica Acta (BBA) - Biomembranes 1758: 1529-1539.
5. Strömstedt AA, Wessman P, Ringstad L, Edwards K, Malmsten M (2007) Effect of lipid headgroup composition on the interaction between melittin and lipid bilayers. Journal of Colloid and Interface Science 311: 59-69.
6. Christensen B, Fink J, Merrifield RB, Mauzerall D (1988) Channel-forming properties of cecropins and related model compounds incorporated into planar lipid membranes. Proceedings of the National Academy of Sciences 85: 5072-5076.
7. Kozlov SA, Vassilevski AA, Feofanov AV, Surovoy AY, Karpunin DV, et al. (2006) Latarcins, Antimicrobial and Cytolytic Peptides from the Venom of the Spider Lachesana tarabaevi (Zodariidae) That Exemplify Biomolecular Diversity. Journal of Biological Chemistry 281: 20983-20992.
8. Dubovskii PV, Volynsky PE, Polyansky AA, Chupin VV, Efremov RG, et al. (2006) Spatial structure and activity mechanism of a novel spider antimicrobial peptide. Biochemistry 45: 10759-10767.
9. Vorontsova OV, Egorova NS, Arseniev AS, Feofanov AV (2011) Haemolytic and cytotoxic action of latarcin Ltc2a. Biochimie 93: 227-241.
10. Corzo G, Villegas E, Gómez-Lagunas F, Possani LD, Belokoneva OS, et al. (2002) Oxyopinins, Large Amphipathic Peptides Isolated from the Venom of the Wolf Spider Oxyopes kitabensis with Cytolytic Properties and Positive Insecticidal Cooperativity with Spider Neurotoxins. Journal of Biological Chemistry 277: 23627-23637.

11. Dubovskii PV, Vassilevski AA, Samsonova OV, Egorova NS, Kozlov SA, et al. (2011) Novel lynx spider toxin shares common molecular architecture with defense peptides from frog skin. FEBS Journal 278: 4382-4393.
12. Pukala TL, Boland MP, Gehman JD, Kuhn-Nentwig L, Separovic F, et al. (2007) Solution structure and interaction of cupiennin 1a, a spider venom peptide, with phospholipid bilayers. Biochemistry 46: 3576-3585.
13. Pukala TL, Doyle JR, Llewellyn LE, Kuhn‐Nentwig L, Apponyi MA, et al. (2007) Cupiennin 1a, an antimicrobial peptide from the venom of the neotropical wandering spider Cupiennius salei, also inhibits the formation of nitric oxide by neuronal nitric oxide synthase. FEBS Journal 274: 1778-1784.
14. Arbuzova A, Schwarz G (1999) Pore-forming action of mastoparan peptides on liposomes: a quantitative analysis. Biochimica et Biophysica Acta (BBA) - Biomembranes 1420: 139-152.
15. Gehman JD, Luc F, Hall K, Lee T-H, Boland MP, et al. (2008) Effect of Antimicrobial Peptides from Australian Tree Frogs on Anionic Phospholipid Membranes. Biochemistry 47: 8557-8565.
16. Kim HJ, Kim SS, Lee MH, Lee BJ, Ryu PD (2004) Role of C‐terminal heptapeptide in pore‐forming activity of antimicrobial agent, gaegurin 4. The Journal of Peptide Research 64: 151-158.
17. Verly RM, Moraes CMd, Resende JM, Aisenbrey C, Bemquerer MP, et al. (2009) Structure and Membrane Interactions of the Antibiotic Peptide Dermadistinctin K by Multidimensional Solution and Oriented 15N and 31P Solid-State NMR Spectroscopy. Biophysical Journal 96: 2194-2203.
18. Pouny Y, Rapaport D, Mor A, Nicolas P, Shai Y (1992) Interaction of antimicrobial dermaseptin and its fluorescently labeled analogs with phospholipid membranes. Biochemistry 31: 12416-12423.
19. Yang L, Weiss TM, Lehrer RI, Huang HW (2000) Crystallization of antimicrobial pores in membranes: magainin and protegrin. Biophysical Journal 79: 2002-2009.
20. Imura Y, Choda N, Matsuzaki K (2008) Magainin 2 in Action: Distinct Modes of Membrane Permeabilization in Living Bacterial and Mammalian Cells. Biophysical Journal 95: 5757-5765.
21. Lee DG, Kim PI, Park Y, Park S-C, Woo E-R, et al. (2002) Antifungal mechanism of SMAP-29 (1–18) isolated from sheep myeloid mRNA against Trichosporon beigelii. Biochemical and Biophysical Research Communications 295: 591-596.
22. Yamaguchi S, Huster D, Waring A, Lehrer RI, Kearney W, et al. (2001) Orientation and dynamics of an antimicrobial peptide in the lipid bilayer by solid-state NMR spectroscopy. Biophysical Journal 81: 2203-2214.
23. Oren Z, Lerman JC, Gudmundsson GH, Agerberth B, Shai Y (1999) Structure and organization of the human antimicrobial peptide LL-37 in phospholipid membranes: relevance to the molecular basis for its non-cell-selective activity. Biochemical Journal 341: 501-513.
24. Lee C-C, Sun Y, Qian S, Huang Huey W (2011) Transmembrane Pores Formed by Human Antimicrobial Peptide LL-37. Biophysical Journal 100: 1688-1696.
25. Gutsmann T, Hagge SO, Larrick JW, Seydel U, Wiese A (2001) Interaction of CAP18-Derived Peptides with Membranes Made from Endotoxins or Phospholipids. Biophysical Journal 80: 2935-2945.
26. Saravanan R, Bhattacharjya S (2011) Oligomeric structure of a cathelicidin antimicrobial peptide in dodecylphosphocholine micelle determined by NMR spectroscopy. Biochimica et Biophysica Acta (BBA) - Biomembranes 1808: 369-381.
27. Subbalakshmi C, Krishnakumari V, Sitaram N, Nagaraj R (1998) Interaction of indolicidin, a 13-residue peptide rich in tryptophan and proline and its analogues with model membranes. Journal of Biosciences 23: 9-13.
28. Zhao H, Mattila J-P, Holopainen JM, Kinnunen PKJ (2001) Comparison of the Membrane Association of Two Antimicrobial Peptides, Magainin 2 and Indolicidin. Biophysical Journal 81: 2979-2991.

29. Gamen S, Hanson DA, Kaspar A, Naval J, Krensky AM, et al. (1998) Granulysin-Induced Apoptosis. I. Involvement of at Least Two Distinct Pathways. The Journal of Immunology 161: 1758-1764.
30. Yoshida K, Mukai Y, Niidome T, Takashi C, Tokunaga Y, et al. (2001) Interaction of pleurocidin and its analogs with phospholipid membrane and their antibacterial activity. Journal of Peptide Research 57: 119-119.
31. Campagna S, Saint N, Molle G, Aumelas A (2007) Structure and Mechanism of Action of the Antimicrobial Peptide Piscidin. Biochemistry 46: 1771-1778.
32. Verdon J, Falge M, Maier E, Bruhn H, Steinert M, et al. (2009) Detergent-Like Activity and [alpha]-Helical Structure of Warnericin RK, an Anti-Legionella Peptide. Biophysical Journal 97: 1933-1940.
33. Raghunathan G, Seetharamulu P, Brooks BR, Guy HR (1990) Models of δ - hemolysin membrane channels and crystal structures. Proteins: Structure, Function, and Bioinformatics 8: 213-225.
34. Park S-C, Kim M-H, Hossain MA, Shin SY, Kim Y, et al. (2008) Amphipathic α-helical peptide, HP (2–20), and its analogues derived from Helicobacter pylori: Pore formation mechanism in various lipid compositions. Biochimica et Biophysica Acta (BBA) - Biomembranes 1778: 229-241.
35. Yang L, Weiss TM, Harroun TA, Heller WT, Huang HW (1999) Supramolecular Structures of Peptide Assemblies in Membranes by Neutron Off-Plane Scattering: Method of Analysis. Biophysical Journal 77: 2648-2656.
36. Shai Y, Bach D, Yanovsky A (1990) Channel formation properties of synthetic pardaxin and analogues. Journal of Biological Chemistry 265: 20202-20209.
37. Vad BS, Bertelsen K, Johansen CH, Pedersen JM, Skrydstrup T, et al. (2010) Pardaxin Permeabilizes Vesicles More Efficiently by Pore Formation than by Disruption. Biophysical Journal 98: 576-585.
