# Supplementary material for: Activity Determinants of Helical Antimicrobial Peptides: A Large-Scale Computational Study
Source: PLoS One. 2013 Jun 12;8(6):e66440. doi: 10.1371/journal.pone.0066440 (PMC3680375; doi:10.1371/journal.pone.0066440)
Supplement: Table S6 — Transfer energy from planar membrane to pores. (PDF) [file pone.0066440.s007.pdf]

Table S6. Transfer energy from planar membrane to pores.
cyl: cylindrical pores. tor: toroidal pores.

Peptide	ΔΔW(cyl-planar)			ΔΔW(tor-planar)		
	30% Anionic	10% Anionic	Neutral	30% Anionic	10% Anionic	Neutral
**1amt**	-3.3±1.3	-3.1±1.5	-2.4±0.5	-4.4±0.5	-3.1±0.9	-3.4±0.3
**1d7n**	1.3±0.6	-1.7±1.0	-0.7±1.2	-2.3±0.8	-3.9±0.9	-2.7±1.6
**1f0d**	1.9±1.5	2.8±1.0	-0.8±2.5	-2.8±2.7	-0.8±2.3	-2.8±2.0
**1f0e**	2.0±2.2	2.7±2.9	-0.5±3.4	-3.7±2.3	-3.4±2.1	-3.6±3.0
**1f0f**	1.7±1.9	2.6±0.8	-3.6±0.5	-5.0±2.0	-2.4±1.0	-5.4±0.9
**1fry**	3.7±2.5	2.6±4.0	-2.4±4.7	-3.7±4.3	-4.2±3.0	-2.2±3.7
**1hu5**	3.4±5.2	-0.5±1.1	-1.1±1.9	-6.3±3.7	-1.8±7.7	-6.8±1.2
**1hu6**	1.0±2.4	-1.0±1.6	-1.2±2.5	-4.9±1.4	-5.1±1.4	-5.1±2.6
**1hu7**	2.0±2.0	-0.4±1.2	-2.6±2.9	-4.9±2.0	-5.2±1.4	-6.3±2.5
**1kv4**	4.5±2.8	1.5±3.3	0.2±1.3	-4.7±2.7	-1.6±6.8	-5.6±1.7
**1lyp**	13.8±1.2	12.1±1.8	3.1±1.2	-1.3±1.8	-0.4±2.3	-7.5±1.6
**1o53**	-6.6±1.3	1.1±0.8	-2.8±1.2	-2.6±0.6	-1.9±0.8	-4.8±0.7
**1p0g**	3.6±1.3	1.2±2.4	-0.2±1.8	-6.3±0.8	-2.8±1.3	-4.1±1.5
**1p0j**	2.7±3.0	-0.6±1.2	-2.4±0.4	-5.7±1.3	-5.0±0.6	-5.2±0.4
**1p0l**	1.7±2.8	-0.5±0.9	-1.9±0.8	-5.3±2.1	-4.9±0.7	-5.2±0.7
**1p0o**	2.1±2.5	-2.1±1.1	-0.2±1.7	-7.3±1.6	-6.0±0.3	-6.1±1.3
**1t51**	-1.8±1.8	0.9±1.7	-1.5±1.2	-1.0±0.8	-1.5±1.5	-2.7±1.5
**1t52**	2.6±0.8	2.0±1.0	-0.9±1.0	-2.2±1.3	-2.4±0.9	-3.7±0.9
**1t54**	1.5±0.9	0.5±1.0	-1.4±1.4	-2.6±0.3	-1.9±1.4	-2.8±0.9
**1t55**	6.1±1.3	1.7±0.3	-1.9±1.4	-1.5±1.2	-0.9±0.8	-3.2±1.0
**1vm2**	-1.7±1.1	-0.4±0.5	-2.1±0.4	-4.2±0.5	-2.5±0.5	-4.4±0.3
**1vm3**	-0.1±0.3	-2.0±1.0	-2.1±0.4	-3.1±0.3	-2.9±0.8	-4.2±0.4
**1vm4**	-0.6±0.5	-1.5±0.7	-2.7±0.5	-3.8±0.5	-3.9±1.1	-4.2±0.8
**1vm5**	-1.3±0.4	-1.0±0.9	-1.9±0.8	-3.4±0.4	-2.5±0.6	-3.8±0.8
**1xc0**	11.3±2.3	1.2±2.5	3.3±2.1	-3.3±2.7	1.9±2.8	2.1±1.7
**1xkm**	-8.2±3.5	4.3±2.1	-1.8±2.0	-2.1±2.9	-0.1±1.7	-4.0±1.8
**1z64**	4.4±1.6	2.6±2.2	-1.2±1.9	-1.2±0.5	0.0±0.7	-2.0±0.9
**1zrw**	-1.4±4.3	-1.3±4.0	-1.6±4.2	-6.4±5.5	-11.1±3.4	-5.0±4.1
**1zrx**	1.5±1.8	3.3±1.3	-1.9±1.7	-3.0±2.8	-1.3±3.1	-2.5±2.1
**2amn**	4.9±2.9	-0.2±2.9	-2.4±4.2	-4.1±3.8	-4.5±3.6	-6.0±1.7
**2czp**	-4.9±5.0	1.1±0.5	-2.1±0.9	-1.1±0.7	-2.0±0.6	-3.6±1.1
**2f3a**	3.1±1.2	0.7±0.9	-1.0±1.6	-5.3±1.4	-2.3±0.8	-4.3±0.9
**2fbs**	1.5±0.9	0.0±1.8	-2.4±1.2	-5.4±0.5	-4.1±1.2	-4.5±1.3
**2g9l**	6.4±2.6	3.4±1.2	1.1±1.3	-4.3±4.2	-1.6±2.3	-3.4±1.4
**2g9p**	1.5±1.2	0.9±3.1	0.7±4.1	-4.4±1.0	-3.9±2.3	-3.7±1.5

Continued from previous page

Peptide	ΔΔW(cyl-planar)			ΔΔW(tor-planar)		
	30% Anionic	10% Anionic	Neutral	30% Anionic	10% Anionic	Neutral
**2gdl**	0.7 ±3.1	2.4 ±3.8	-2.5 ±2.7	-1.8 ±3.5	-0.7 ±5.1	-3.1 ±2.9
**2hfr**	6.6 ±3.7	-2.8 ±3.5	-2.8 ±2.3	-4.5 ±4.3	-4.3 ±3.8	-3.1 ±2.1
**2jmy**	0.4 ±0.9	2.4 ±1.1	-1.8 ±1.1	-4.7 ±0.4	-3.9 ±0.6	-4.2 ±0.9
**2jos**	1.2 ±2.8	0.8 ±3.2	1.8 ±2.0	-3.0 ±0.9	-2.2 ±1.3	-4.1 ±0.7
**2jpy**	0.0 ±1.1	-1.6 ±0.6	-0.8 ±1.0	-2.9 ±1.0	-1.4 ±0.6	-3.5 ±0.6
**2jq0**	-3.3 ±0.7	-0.5 ±1.8	-1.8 ±1.0	-2.8 ±1.1	-2.0 ±0.9	-3.1 ±2.4
**2jq1**	-1.0 ±0.9	-0.8 ±1.6	-1.3 ±1.1	-2.6 ±1.1	-2.1 ±0.8	-4.8 ±0.8
**2jr8**	7.1 ±2.6	4.2 ±3.3	-2.6 ±1.1	-2.5 ±1.4	-3.5 ±2.2	-6.2 ±1.7
**2k10**	6.8 ±2.0	3.0 ±1.1	-0.5 ±5.4	-3.1 ±1.2	-2.4 ±0.5	-4.5 ±1.3
**2k38**	0.6 ±1.7	2.9 ±3.1	-2.0 ±1.1	-0.7 ±3.9	-2.5 ±3.3	-4.0 ±2.6
**2k6o**	8.8 ±1.4	7.3 ±2.7	2.4 ±3.5	-2.9 ±2.2	-3.8 ±4.1	-1.0 ±4.6
**2k9b**	1.3 ±3.0	3.7 ±1.5	-1.6 ±2.5	-1.7 ±1.4	-2.3 ±1.6	-3.6 ±2.6
**2kam**	6.2 ±0.8	-3.0 ±1.8	-1.0 ±2.0	-5.5 ±1.8	-3.0 ±1.5	-2.3 ±0.9
**2l3i**	3.5 ±4.7	5.8 ±3.6	0.7 ±3.2	-6.0 ±3.1	-1.0 ±4.4	-4.5 ±2.9
**2lmf**	-4.3 ±2.9	2.5 ±1.4	-0.3 ±2.2	-2.8 ±2.0	-1.7 ±3.4	-3.8 ±3.1
**2mag**	3.6 ±1.9	0.6 ±1.3	-3.1 ±2.4	-3.5 ±2.8	-2.8 ±1.4	-6.0 ±0.7
**2mlt**	9.3 ±2.0	-2.7 ±2.2	-0.5 ±3.3	-5.4 ±1.2	-5.3 ±2.5	-3.0 ±3.0
**2pco**	-2.6 ±2.2	0.1 ±3.3	-2.8 ±1.9	-4.6 ±3.1	-7.1 ±3.0	-6.6 ±1.5
