# Supplementary material for: Activity Determinants of Helical Antimicrobial Peptides: A Large-Scale Computational Study
Source: PLoS One. 2013 Jun 12;8(6):e66440. doi: 10.1371/journal.pone.0066440 (PMC3680375; doi:10.1371/journal.pone.0066440)
Supplement: Table S7 — Hydrophobic quadrupole moment of studied peptides. (PDF) [file pone.0066440.s008.pdf]

Table S7. Hydrophobic quadrupole moment of studied peptides.

$<Q>_{res}$: hydrophobic quadrupole moment per residue; Q : total hydrophobic quadrupole moment.

PDBid	$<Q>_{res}$	Q	PDBid	$<Q>_{res}$	Q
**1amt**	59.51	1190.25	**1zrw**	34.04	851.07
**1d7n**	39.39	551.49	**1zrx**	63.48	2665.96
**1f0d**	56.44	1128.79	**2amn**	117.88	3064.78
**1f0e**	72.91	1458.29	**2czp**	28.06	392.84
**1f0f**	66.45	1196.15	**2f3a**	37.79	491.26
**1fry**	41.06	1190.80	**2fbs**	24.59	319.66
**1hu5**	71.88	1293.85	**2g9l**	106.60	3944.05
**1hu6**	27.18	489.18	**2g9p**	85.03	2210.85
**1hu7**	95.36	1716.53	**2gdl**	31.36	972.27
**1kv4**	269.67	11326.10	**2hfr**	246.98	6668.33
**1lyp**	124.99	3999.66	**2jmy**	20.74	311.04
**1o53**	44.18	574.34	**2jos**	84.77	1864.98
**1p0g**	105.69	2008.17	**2jpy**	103.67	1969.73
**1p0j**	70.72	1343.68	**2jq0**	50.27	955.06
**1p0l**	78.89	1498.99	**2jq1**	93.30	1772.69
**1p0o**	50.27	955.14	**2jr8**	132.16	5550.72
**1t51**	70.31	914.07	**2k10**	74.04	1480.80
**1t52**	57.71	750.19	**2k38**	103.33	3306.56
**1t54**	32.65	424.39	**2k6o**	97.26	3598.73
**1t55**	52.03	676.41	**2k9b**	160.50	5296.50
**1vm2**	36.90	479.73	**2kam**	55.20	1435.20
**1vm3**	42.14	547.82	**2l3i**	67.23	2016.89
**1vm4**	41.98	545.71	**2lmf**	34.01	782.28
**1vm5**	43.68	567.79	**2mag**	66.62	1532.31
**1xc0**	138.83	4581.39	**2mlt**	77.36	2011.47
**1xkm**	81.43	1791.46	**2pco**	67.31	1750.01
**1z64**	101.92	2548.07			
