# Supplementary material for: Activity Determinants of Helical Antimicrobial Peptides: A Large-Scale Computational Study
Source: PLoS One. 2013 Jun 12;8(6):e66440. doi: 10.1371/journal.pone.0066440 (PMC3680375; doi:10.1371/journal.pone.0066440)
Supplement: Section S1 — Standardization of the membrane binding free energies. (PDF) [file pone.0066440.s009.pdf]

Supporting Information: Activity determinants of helical antimicrobial peptides

*Yi He and Themis Lazaridis**

* Department of Chemistry, City College of New York, New York, 10021, U.S.A, Tel: (212) 650-8364, E-mail: tlazaridis@ccny.cuny.edu

S1 Standardization of the membrane binding free energies

The standard membrane binding free energy can be defined based on a number of different equilibrium constants. One is based on the peptide molarities in the lipid $[P_L]$ and aqueous phase $[P_w]$ [1]:

$$\Delta G_c^0 = -RTlnK_c, where\ K_c = \frac{[P_L]}{[P_w]} \quad (1)$$

where $[P_L]$ is defined as moles of peptide per volume of the lipid phase for membrane-inserting peptides or per volume of the interfacial region for interfacially adsorbed peptides. An alternative definition of the standard free energy is based on the mole fraction partition coefficient K_x:

$$\Delta G_x^0 = -RTlnK_x, where\ K_x = \frac{n_b/(n_b + n_L)}{n_f/(n_f + n_w)} \quad (2)$$

in which n_b, n_f, n_L and n_w are the moles of bound peptide, free peptide, lipid and water molecules, respectively . Because under most experimental conditions, $n_b << n_L$ and $n_f << n_w$, K_x can be approximated as:

$$K_x \approx \frac{n_b/n_L}{n_f/n_w} \quad (3)$$

ΔG_x^0 can be converted to ΔG_c^0 as follows[1]:

$$\Delta G_c^0 = -RTln\frac{[P_L]}{[P_w]} = -RTln\frac{n_b/V_L}{n_w/V_w} = -RTln\frac{n_b/n_L v_L}{n_f/n_w v_w}$$

$$= -RTln\frac{n_b/n_L}{n_f/n_w} + RTln\frac{v_L}{v_w}$$

$$\cong \Delta G_x^0 + RTln\frac{v_L}{v_w} \quad (4)$$

where V_L and V_w are the total volume of the lipid and water, v_w and v_L are the molar volumes of water (0.018 M^{-1}) and lipid (0.76 M^{-1} , based on DOPC volume [2]) respectively. At room temperature (298 K), used here and in most experiments, the term $RTln(v_L/v_w)$ is equal to 2.2 kcal/mol.

K_x can be obtained by measuring the fraction of peptide bound to the membrane f_b ($f_b = n_b/(n_b + n_f)$) at different lipid concentrations [L]:

$$f_b = \frac{n_b}{n_b + \frac{n_b/n_L}{K_x/n_w}} = \frac{K_x n_L}{K_x n_L + n_w} = \frac{K_x[L]}{K_x[L] + [W]} \quad (5)$$

where [W] is the water concentration, close to 55.3 M at room temperature.

Another definition of the binding free energy is based on the association or dissociation constant (K_a or K_d) of peptide to membrane. So:

$$P + L \rightarrow PL \quad (6)$$

$$K_d = \frac{1}{\mathrm{K_a}} = \frac{[\mathrm{P}_f][\mathrm{L}]}{[\mathrm{PL}]} \quad (7)$$

$$\Delta G_a^0 = -RTlnK_a = RTlnK_d \ (8)$$

where $[P_f]$ and [PL] are the concentration of free peptides and peptide/lipid complexes over the entire volume of the system. The definition of [L] is a little ambiguous: because of the collective behavior of the lipids, the membrane should be treated as an ensemble of lipids[3]. Here, we assume the binding sites are lipid ensembles composed of *n* lipid molecules on average. [L] is thus defined as the concentration of binding sites $[L_n]$. Because the lipid concentration is normally low, $V_{system} \approx V_w$, thus $[P_f]$ is close to $[P_w]$. So:

$$\Delta G_c^0 = -RTln\frac{[P_L]}{[P_w]} \approx -RTln\frac{n_b/V_L}{[P_f]} = -RTln\frac{[PL_n]V_{system}/V_L}{[P_f]} = -RTln\frac{V_{system}/V_L}{K_d/[L_n]} = RTlnK_d v_L' = \Delta G_a^0 + RTlnv_L' \quad (9)$$

where v'_L is the molar volume of the lipid binding site. K_d can be measured from binding kinetics or by fitting the titration curve to a binding model (see below). In most papers reporting K_d, v'_L is treated as v_L. Because a lipid binding site can contain up to 10-20 lipids [4-6], neglecting this can result in an energy difference of 1.36 to 1.77 kcal/mol.

K_d can be obtained from binding kinetics[7] or by one-site model [8]. In the latter, lipid membrane is assumed to be a single site receptor that can be saturated with enough peptides and the concentration-independent dissociation constant K_d can be calculated from fitting the following equation:

$$R_b = \frac{R_{max}[P_w]}{K_d + [P_w]} \quad (10)$$

R_b is the molar ratio of bound peptide in the lipid ($R_b = n_b/n_L$). R_{max} is the maximum capacity of the binding site (R_{max}= 1/n). Equation 10 can be transformed into:

$$K_d == \frac{(1-f_b)[L]}{nf_b} - [P](1-f_b) \; (11)$$

if the fraction of bound peptide f_b ($f_b = n_b/(n_f+n_b)$) is measured. In this equation, [P] and [L] is the total concentration of peptide and lipid respectively.

In this paper we adopt ΔG_c because of two main advantages over ΔG_x and ΔG_a. First, molarities arise naturally in statistical thermodynamic treatments (see below) and second, it is independent of the relative molecular size of the lipid and water, as it should intuitively. One disadvantage of ΔG_c is that the definition of the volume of the lipid phase V_L is somewhat arbitrary and differs for membrane-inserting and adsorbed peptides.

Alternative equilibrium constants can be found in the literature, for example[9]:

$$K_{app} = R_b/[P_w] \; (12)$$

K_{app} can be used to calculate ΔG_c^0:

$$\Delta G_c^0 = -RTln\frac{[P_L]}{[P_w]} = -RTln\frac{n_b/V_L}{[P_w]}$$

$$= -RTln\frac{R_b n_L/V_L}{[P_w]}$$

$$= -RTln\frac{K_{app}}{v_L}$$

$$= \Delta G_{app}^0 + RTlnv_L \quad (13)$$

One disadvantage of K_{app} is that K_{app} is not constant but changes with peptide concentration[10]. This is because the peptide could saturate the membrane and prevent further binding; when peptides get too crowded on the membrane, the electrostatic repulsion between charged antimicrobial peptides becomes significant. For anionic membranes, the saturation happens easier because the higher affinity of peptide to the membrane. To obtain a concentration-independent partition coefficient, many papers maintained the peptide to lipid ratio at a very low value so that the peptide-peptide interaction is negligible. This makes experimental measurements more difficult. To allow a wider concentration range, some authors removed the electrostatic interaction component from the binding free energy [10-12] using the following method.

Because of the electrostatic interaction between the membrane and peptide, the peptide concentration in the aqueous solution immediately adjacent to the membrane $[P_m]$ is:

$$[P_m] = [P_w] exp\left(-zF_0 \frac{\phi_0}{RT}\right) \quad (14)$$

where z is the charge of the peptide, F_0 is the Faraday constant, and ϕ_0 is the electrostatic potential on the membrane surface, which can be obtained from the Gouy-Chapman theory:

$$\sigma^2 = 2000\epsilon_0\epsilon_R RT \sum_i C_{i,eq} \left(e^{-\frac{z_i F_0 \phi_0}{RT}} - 1\right) \quad (15)$$

where $\epsilon_0\epsilon_R$ is the dielectric constant, $C_{i,eq}$ is the concentration of each ion species in the solution, z_i is the charge of each ion species. σ is the charge density on the membrane surface, defined as a sum of charges on anionic lipids and on surface bound peptides (e_o is the unit charge, A_L and A_p are the area of lipid and peptide molecules, X_{PG} is the fraction of anionic lipid):

$$\sigma = \frac{(e_0/A_L)(-X_{PG} + X_b z_p)}{\left[1 + X_b\left(\frac{A_p}{A_L}\right)\right]} \quad (16)$$

Using $[P_m]$, a partition coefficient K'_{app} that is independent of peptide concentration can be obtained:

$$K'_{app} = \frac{R_b}{[P_m]} \quad (17)$$

However, the binding energy $\Delta G'_c$ calculated from K'_{app} does not include the electrostatic interaction between peptide and anionic lipids, which makes it unsuitable for our purposes. We corrected such reported binding free energies by adding back the electrostatic interaction energy between peptide and membrane:

$$\Delta G_c^0 = \Delta G'_c + z\phi \quad (18)$$

where z is the effective charge of the peptide and φ is the membrane surface potential calculated from Gouy-Chapman theory.

The following experimental methods are commonly used to determine the binding energy:

1. CD titration. In CD experiments[13,14], the moles of bound peptide n_b can be calculated from the change in ellipticity θ because of conformational change upon peptide transfer from water solution to the membrane. By titrating a small amount of peptide solution into the lipid solution or a small amount of liposome solution into the peptide solution, the binding isotherm can be established. Or, the fraction of bound peptide f_b can be obtained and used to calculate K_x or K_d using equation 5 and 18.
2. Isothermal titration calorimetry (ITC). Small amounts of peptide are injected into liposome solutions periodically (or reversely, liposomes are titrated into a peptide solution)[11,15]. Heat is generated in the binding reaction. The number of membrane-bound molecules is calculated from the molar binding enthalpy and is used to calculate the molar ratio of bound peptide R_b. The binding free energy can be calculated using the same method as CD experiments.
3. Fluorescence titration. Another method used to obtain the binding isotherm is to measure the fluorescence intensity of a tryptophan residue on the peptide or fluorophore labels attached to peptide sidechains[16,17]. The molar ratio of bound peptide R_b can be calculated from the fluorescence intensity. R_b can be used to further calculate K_d using equation 17.

4. Fluorescence kinetics. In this method, the resonance between a tryptophan residue on the peptide and a fluorescent probe in the membrane indicates the extent of peptide binding. The rate of peptide association (k_{on}[L]) and dissociation (k_{off}) to membrane surface can be measured[7]:

$$P_w \underset{k_{off}}{\overset{k_{on}[L_n]}{\leftrightharpoons}} P_L \quad (19)$$

One has to note that [L] is used in the literature instead of [L_n] so the reported K_d is actually nK_d. The fluorescence intensity can be expressed as:

$$F(t) = a_0\left[1 - \exp(-k_{app}t)\right] + a_1 \quad (20)$$

where a_0 and a_1 are constant and the apparent rate constant k_{app} is the sum of both directions:

$$k_{app} = k_{on}[L_n] + k_{off} \quad (21)$$

k_{app} can be obtained by fitting the kinetic curves. By changing the lipid concentration, the rate constant k_{on} and k_{off} can be determined. The dissociation constant K_d can be calculated as:

$$K_d = \frac{k_{off}}{k_{on}} \quad (22)$$

K_d can be used to calculate the binding free energy using the relationship shown above. The binding free energy calculated from the reported K_d is:

$$\Delta G_c^0 = RTlnK_d v_L \quad (23)$$

5. Surface plasmon resonance. Another way to measure binding kinetics is surface plasmon resonance[18]. By injecting peptide solution to a membrane surface illuminated by surface plasmon polaritons, we can obtain the change in surface response with time during the association process. The kinetics of association is assumed to be two steps: 1) fast association with lipid. 2) peptide insertion into the membrane to form a tighter complex.

$$P_w + L_n \underset{k_{d1}}{\overset{k_{a1}}{\leftrightharpoons}} P_L \underset{k_{d2}}{\overset{k_{a2}}{\leftrightharpoons}} PL^* \quad (24)$$

The rate constants k_{a1}, k_{a2}, k_{d1}, k_{d2} can be calculated from the following differential equations:

$$\frac{dR_1}{dt} = k_{a1}[P](R_{max} - R_1 - R_2) - k_{d1}R_1 - k_{a2}R_1 + k_{d2}R_2 \quad (25)$$

$$\frac{dR_2}{dt} = k_{a2}R_1 - k_{d2}R_2 \quad (26)$$

where R_1, R_2, R_{max} are the surface response contributed by peptide lipid complex produced by first step, second and 100% peptide-lipid complex respectively. The dissociation constant of step 1 and step 2 are k_{d1}/k_{a1} and k_{d2}/k_{a2} respectively. The overall dissociation constant is the product of K_{d1} and K_{d2} . The overall dissociation constant can be used to calculate the binding free energy using equation 8.

6. Ultrafiltration or Reverse HPLC. The peptide is mixed with lipid for sufficient time to allow full binding. Then the solution is centrifuged through a filter[19] or run through HPLC[20] where membrane and free peptide solution can be separated. The peptide concentration that remained in solution and bound to the membrane thus can be determined. In this method, the partition coefficient K_p is defined as:

$$K_p = \frac{[P_b]/v_w}{[P_f]/v_L} \quad (27)$$

Thus the binding free energy can be calculated as:

$$\Delta G_c^0 = -RTlnK_p + RTln\frac{v_L}{v_w} \quad (28)$$

7. Tryptophan time-resolved fluorescence. Because the life-time (τ) of tryptophan fluorescent excited by a laser pulse is dependent on the surrounding environment, the τ measured in peptide-lipid mixtures has the following relationship with the fluorescence life time τ_w in water and τ_L in lipid[21]:

$$\tau = \frac{\tau_w + K_p v_L [L] \tau_L}{1 + K_p v_L [L]} \quad (29)$$

where,

$$K_p = \frac{n_b/(n_L v_L)}{n_f/(n_w v_w)} \quad (30)$$

K_p can be obtained by fitting the above equation under different lipid concentration. Based on the definition, K_p is actually K_c. Thus the binding free energy can be calculated as:

$$\Delta G_c^0 = -RTlnK_p \quad (31)$$

8. EPR. The resonance intensity can be used to calculate the bound peptide ratio $\lambda = n_b/n_f$. By titrating lipid into peptide solution, the partition coefficient K_c can be calculated from fitting the following equation[22]:

$$\frac{1}{\lambda} = \frac{1}{K_c v_L'}\left(\frac{1}{[L]} - v_L\right) \quad (32)$$

in the above equation, v_L' is the effective molar volume available for peptide and can be defined as:

$$v_L' = \frac{A_L(d/2)}{n_L N_a} \quad (33)$$

The obtained binding free energy is ΔG_c.

For all of the above experiments, because not all lipids of the vesicle are available for binding, the actual peptide/lipid ratio R_b^* is higher than the measured value R_b. For SUV, the outer leaflet is about 60% of the total lipids, so the R_b^* should be $R_b/0.6$.The binding energy is 0.30 kcal/mol lower if this is not considered. This correction may not be valid if the peptide can translocate across the membrane. Unless there is strong evidence for translocation, we added 0.30 kcal/mol to the binding free energy values when this effect had not already been corrected in the experiments.

The binding energy can be estimated using several theoretical methods:

1. Hydrophobicity scale

An empirical way to calculate the membrane binding free energy is to use a hydrophobic scale. The free energy contribution of transferring each amino acid from water to the POPC interface was determined by Wimley and White[23]. The transfer free energy of a peptide can thus be calculated as the sum of the contributions from all residues. This method utilizes the peptide sequence and thus neglects the effects of secondary and tertiary structure. The calculated values are not always in agreement with the measured binding free energy[24]. In this method the reported binding free energy is ΔG_x.

2. Binding free energy from the potential of mean force

Umbrella sampling simulations can produce the binding free energy as a function of a reaction coordinate in MD simulations. The binding free energy is calculated by integration of the PMF curves [25,26]:

$$\Delta G_c^0 = RTln\frac{[P_L]}{[P_w]} = -RTln\left[\frac{1}{d}\int_0^d dze^{-\frac{W(z)}{k_bT}}\right] \quad (34)$$

where z is the distance of the peptide center of mass from the membrane center and *d* is a cutoff for z that defines the membrane-bound state. A similar method was originallly proposed by Ben-Tal et al.[27]:

$$\Delta G = -\mathrm{RTlnK}$$
$$where\ K = CA_L N_A \int_0^{\infty} dz(e^{-\frac{W(z)}{k_b T}} - 1)\ (35)$$

A_L is the area per lipid, N_A is the Avogadro constant, C is a unit conversion factor. In this equation, the K is actually K_x/[W], so the $\Delta G_c^0 = -\mathrm{RTlnK} + RTlnv_L = \Delta G + RTlnv_L$.

This method was used to compute the binding free energy of lactoferricin to POPC[25] and POPG membrane [26], the binding free energy of protegrin to POPE/POPG(3:1) bilayers[28], the binding free energy of indolicidin to DMPC and DMPC/DMPG(3:1) bilayers[29], and the binding free energy of melittin to POPC bilayers[30] .

3. Simpler theoretical approaches

Starting from Eq. 35 one can express the standard free energy as the sum of the average effective energy relative to the bulk (<W>) and terms of the form ∫plnp corresponding to translational and rotational entropy[31,32]. The entropic terms have been found to be rather small[33] . This allows a faster estimate of the membrane binding free energy by simple implicit solvent simulations without the need to compute the potential of mean force. This approach was followed in a calculation of pH-dependent membrane binding free energies[34].

Reference:

1. White SH, Wimley WC (1999) MEMBRANE PROTEIN FOLDING AND STABILITY: Physical Principles. Annual Review of Biophysics and Biomolecular Structure 28: 319-365.
2. Chiu SW, Jakobsson E, Subramaniam S, Scott HL (1999) Combined Monte Carlo and Molecular Dynamics Simulation of Fully Hydrated Dioleyl and Palmitoyl-oleyl Phosphatidylcholine Lipid Bilayers. Biophysical Journal 77: 2462-2469.
3. White SH, Wimley WC, Ladokhin AS, Hristova K (1998) [4] Protein folding in membranes: Determining energetics of peptide-bilayer interactions. In: Gary K. Ackers MLJ, editor. Methods in Enzymology: Academic Press. pp. 62-87.
4. Silvestro L, Gupta K, Weiser JN, Axelsen PH (1997) The concentration-dependent membrane activity of cecropin A. Biochemistry 36: 11452-11460.
5. Verly RM, Rodrigues MA, Daghastanli KRP, Denadai AML, Cuccovia IM, et al. (2008) Effect of cholesterol on the interaction of the amphibian antimicrobial peptide DD K with liposomes. Peptides 29: 15-24.
6. Yamamoto N, Tamura A (2010) Designed low amphipathic peptides with alpha-helical propensity exhibiting antimicrobial activity via a lipid domain formation mechanism. Peptides 31: 794-805.
7. Gregory SM, Cavenaugh A, Journigan V, Pokorny A, Almeida PFF (2008) A Quantitative Model for the All-or-None Permeabilization of Phospholipid Vesicles by the Antimicrobial Peptide Cecropin A. Biophysical Journal 94: 1667-1680.
8. Abraham T, Lewis RN, Hodges RS, McElhaney RN (2005) Isothermal titration calorimetry studies of the binding of a rationally designed analogue of the antimicrobial peptide gramicidin s to phospholipid bilayer membranes. Biochemistry 44: 2103-2112.
9. Peitzsch RM, McLaughlin S (1993) Binding of acylated peptides and fatty acids to phospholipid vesicles: Pertinence to myristoylated proteins. Biochemistry 32: 10436-10443.

10. Beschiaschvili G, Seelig J (1990) Peptide binding to lipid bilayers. Binding isotherms and zeta-potential of a cyclic somatostatin analogue. Biochemistry 29: 10995-11000.
11. Wenk MR, Seelig J (1998) Magainin 2 amide interaction with lipid membranes: calorimetric detection of peptide binding and pore formation. Biochemistry 37: 3909-3916.
12. Wieprecht T, Apostolov O, Seelig J (2000) Binding of the antibacterial peptide magainin 2 amide to small and large unilamellar vesicles. Biophysical Chemistry 85: 187-198.
13. Beschiaschvili G, Seelig J (1990) Melittin binding to mixed phosphatidylglycerol/phosphatidylcholine membranes. Biochemistry 29: 52-58.
14. Wieprecht T, Dathe M, Beyermann M, Krause E, Maloy WL, et al. (1997) Peptide Hydrophobicity Controls the Activity and Selectivity of Magainin 2 Amide in Interaction with Membranes. Biochemistry 36: 6124-6132.
15. Wieprecht T, Beyermann M, Seelig J (1999) Binding of Antibacterial Magainin Peptides to Electrically Neutral Membranes: Thermodynamics and Structure. Biochemistry 38: 10377-10387.
16. Pouny Y, Rapaport D, Mor A, Nicolas P, Shai Y (1992) Interaction of antimicrobial dermaseptin and its fluorescently labeled analogs with phospholipid membranes. Biochemistry 31: 12416-12423.
17. Gazit E, Boman A, Boman HG, Shai Y (1995) Interaction of the Mammalian Antibacterial Peptide Cecropin P1 with Phospholipid Vesicles. Biochemistry 34: 11479-11488.
18. Gaidukov L, Fish A, Mor A (2003) Analysis of Membrane-Binding Properties of Dermaseptin Analogues: Relationships between Binding and Cytotoxicity. Biochemistry 42: 12866-12874.
19. Allende D, Vidal A, Simon SA, McIntosh TJ (2003) Bilayer interfacial properties modulate the binding of amphipathic peptides. Chemistry and Physics of Lipids 122: 65-76.
20. Ladokhin AS, Selsted ME, White SH (1997) Bilayer interactions of indolicidin, a small antimicrobial peptide rich in tryptophan, proline, and basic amino acids. Biophysical Journal 72: 794-805.
21. Bastos M, Bai G, Gomes P, Andreu D, Goormaghtigh E, et al. (2008) Energetics and Partition of Two Cecropin-Melittin Hybrid Peptides to Model Membranes of Different Composition. Biophysical Journal 94: 2128-2141.
22. Lewis JR, Cafiso DS (1999) Correlation between the free energy of a channel-forming voltage-gated peptide and the spontaneous curvature of bilayer lipids. Biochemistry 38: 5932-5938.
23. Wimley WC, White SH (1996) Experimentally determined hydrophobicity scale for proteins at membrane interfaces. Nature Structural & Molecular Biology 3: 842-848.
24. Clark KS, Svetlovics J, McKeown AN, Huskins L, Almeida PF (2011) What Determines the Activity of Antimicrobial and Cytolytic Peptides in Model Membranes. Biochemistry 50: 7919-7932.
25. Vivcharuk V, Tomberli B, Tolokh IS, Gray CG (2008) Prediction of binding free energy for adsorption of antimicrobial peptide lactoferricin B on a POPC membrane. Physical Review E 77.
26. Tolokh IS, Vivcharuk V, Tomberli B, Gray CG (2009) Binding free energy and counterion release for adsorption of the antimicrobial peptide lactoferricin B on a POPG membrane. Physical Review E 80.
27. Ben-Tal N, Honig B, Peitzsch RM, Denisov G, McLaughlin S (1996) Binding of small basic peptides to membranes containing acidic lipids: theoretical models and experimental results. Biophysical Journal 71: 561-575.
28. Vivcharuk V, Kaznessis Y (2010) Free Energy Profile of the Interaction between a Monomer or a Dimer of Protegrin-1 in a Specific Binding Orientation and a Model Lipid Bilayer. The Journal of Physical Chemistry B 114: 2790-2797.
29. Yeh I-C, Ripoll DR, Wallqvist A (2012) Free Energy Difference in Indolicidin Attraction to Eukaryotic and Prokaryotic Model Cell Membranes. The Journal of Physical Chemistry B 116: 3387-3396.
30. Irudayam SJ, Berkowitz ML (2012) Binding and reorientation of melittin in a POPC bilayer: Computer simulations. Biochimica et Biophysica Acta (BBA) - Biomembranes 1818: 2975-2981.
31. Lazaridis T, Masunov A, Gandolfo F (2002) Contributions to the binding free energy of ligands to avidin and streptavidin. Proteins 47: 194-208.

32. Lazaridis T, Karplus M (1999) Effective energy function for proteins in solution. Proteins 35: 133-152.
33. Ben-Tal N, Honig B, Bagdassarian CK, Ben-Shaul A (2000) Association Entropy in Adsorption Processes. Biophysical Journal 79: 1180-1187.
34. Mihajlovic M, Lazaridis T (2006) Calculations of pH-Dependent Binding of Proteins to Biological Membranes. The Journal of Physical Chemistry B 110: 3375-3384.
